# Assessing the size of non-Māori-speakers’ active Māori lexicon

**DOI:** 10.1371/journal.pone.0289669

**Published:** 2023-08-23

**Authors:** Yoon Mi Oh, Simon Todd, Clay Beckner, Jen Hay, Jeanette King

**Affiliations:** 1 Department of French Language and Literature, Ajou University, Suwon, Gyeonggi-do, South Korea; 2 Department of Linguistics, University of California, Santa Barbara, California, United States of America; 3 New Zealand Institute of Language, Brain and Behaviour, NZILBB, University of Canterbury, Christchurch, New Zealand; 4 Department of Applied Linguistics, University of Warwick, Coventry, United Kingdom; 5 Department of Linguistics, University of Canterbury, Christchurch, New Zealand; 6 Aotahi—School of Māori and Indigenous Studies, University of Canterbury, Christchurch, New Zealand; The Education University of Hong Kong, HONG KONG

## Abstract

Most non-Māori-speaking New Zealanders are regularly exposed to Māori throughout their lives without seeming to build any extensive Māori lexicon; at best, they know a small number of words which are frequently used and sometimes borrowed into English. Here, we ask how many Māori words non-Māori-speaking New Zealanders know, in two ways: how many can they identify as real Māori words, and how many can they actively define? We show that non-Māori-speaking New Zealanders can readily identify many more Māori words than they can define, and that the number of words they can reliably define is quite small. This result adds crucial support to the idea presented in earlier work that non-Māori-speaking New Zealanders have implicit form-based (proto-lexical) knowledge of many Māori words, but explicit semantic (lexical) knowledge of few. Building on this distinction, we further ask how different levels of word knowledge modulate effects of phonotactic probability on the accessing of that knowledge, across both tasks and participants. We show that participants’ implicit word knowledge leads to effects of phonotactic probability–and related effects of neighbourhood density–in a word/non-word discrimination task, but not in a more explicit task that requires the active definition of words. Similarly, we show that the effects of phonotactic probability on word/non-word discrimination are strong among participants who appear to lack explicit word knowledge, as indicated by their weak discrimination performance, but absent among participants who appear to have explicit word knowledge, as indicated by their strong discrimination performance. Together, these results suggest that phonotactic probability plays its strongest roles in the absence of explicit semantic knowledge.

## Introduction

How many words of Māori can non-Māori-speaking New Zealanders actively define? Despite the fact that the Māori vocabulary size of non-Māori-speaking New Zealanders has been assessed in multiple studies, this is not known. This is because studies tend to focus on identifying words for which the semantics can be passively recognized, rather than more actively produced.

This question is of particular current importance, given an emerging literature that shows that non-Māori-speaking New Zealanders have impressive gradient knowledge of the forms that can and do constitute Māori words. Such knowledge is argued to derive from a *proto-lexicon*–a large collection of implicit memories of the forms of words and word-parts that are experienced with statistical regularity in Māori, without associated semantics. This argument crucially assumes that the implicit proto-lexicon is much larger than, and is thus not constituted by, the explicit vocabulary of Māori words that non-Māori-speaking New Zealanders can actively use. This crucial assumption has not yet been empirically tested. In this paper, we test this assumption empirically for the first time, through two experiments that assess different aspects of Māori vocabulary knowledge amongst non-Māori-speaking New Zealanders.

## Background

### Te reo Māori in New Zealand

Te reo Māori (henceforth referred to as ‘Māori’) is a Polynesian language, and the language of the Indigenous people of New Zealand. It has a small phoneme inventory, consisting of ten consonants /p, t, k, m, n, ŋ, w, f, ɾ, h/ and five vowels /i, e, a, o, u/. All phonemes map to distinct graphemes; /ŋ/ and /f/ map to digraphs (<ng> and <wh>, respectively), while all other phonemes map to single characters. This mapping is bidirectional: there are no silent characters or phonological processes that complicate the mapping from graphemes to phonemes, and there is no ambiguity in the phonological structure of written forms due to a simple syllable template that lacks consonant clusters. The five vowels also have long forms, which are indicated orthographically with a macron over the vowel. The spelling system is transparent and known to New Zealanders, which facilitates the use of written stimuli in experiments designed to tap phonological knowledge.

According to the 2018 New Zealand Census, only approximately 4% of the New Zealand population are able to speak Māori well enough to have an everyday conversation [1]. However, most New Zealanders have some degree of limited knowledge of it. For example, most New Zealanders are familiar with a relatively small vocabulary of loanwords that have been borrowed from Māori into New Zealand English, primarily in the areas of flora and fauna (e.g., *pōhutakawa*, a kind of tree, and *kiwi*, a kind of bird) as well as Māori culture (e.g., *marae*, the complex on which a meeting house is situated, and *haka*, a traditional war dance) [2]. In addition, many New Zealanders learn a handful basic vocabulary items such as numbers, colours, and body parts through early schooling, either explicitly or in the context of songs. These targeted opportunities to actively learn Māori words are augmented by incidental opportunities to passively learn through everyday experiences, as most New Zealanders are regularly exposed to Māori in their daily life through the media and through their social environments [3].

Knowledge of, and exposure to, Māori has been increasing among non-Māori-speaking New Zealanders in recent years, in parallel with the development of positive attitudes toward Māori both societally and individually [4,5]. Māori words are increasingly used by non-Māori-speaking New Zealanders to “[express] solidarity with the Māori perspective and the desire of aligning their identity within a Māori background” [4: 52]. The same patterns are seen in the extent to which the usage of Māori words is faithful to underlying Māori grammar. While, historically, loanwords have been fully adapted to English phonology and morphology, there is a retreat away from this, with speakers signalling positive stance toward Māori by producing more Māori-like phonetics (such as the alveolar tap /ɾ/ rather than the approximant /ɹ/), and by adopting more Māori-like morphological behaviour (such as omitting the English plural marker /z/, as Māori uses a null-plural) [5]. These changes also mean that Māori is increasingly used–in a faithful way–at public events, in schools, and in the media, which increases everyday exposure to Māori and the incidental learning it brings.

### The Māori proto-lexicon

Recent results suggest that incidental learning through everyday exposure endows New Zealanders who do not speak Māori with a ‘proto-lexicon’ of more than a thousand words or word-parts [6,7]. This suggestion is based on experimental results in which non-Māori-speaking New Zealanders demonstrate both lexical knowledge–i.e. the ability to discriminate between Māori words and highly Māori-like non-words–and phonotactic knowledge–i.e. the ability to rate the gradient well-formedness of non-words in an extremely accurate and fine-grained manner, comparable to fluent speakers of the language [6]. Crucially, the demonstrations of these two kinds of knowledge are linked: participants who are better able to discriminate words and non-words also have more sensitivity to phonotactic probabilities [7], implying that phonotactic knowledge is generated as a generalisation over a stored proto-lexicon.

A key assumption of the proto-lexicon claim is that non-Māori-speaking New Zealanders do not have conscious or active knowledge of the meanings of forms in their proto-lexicon. While this assumption is intuitive–New Zealanders who do not speak Māori are highly unlikely to know the meanings of over a thousand Māori words–it is not yet directly supported with empirical evidence. In this paper, we aim to fill this empirical gap by conducting a word identification task and a definition task on a single set of words, to establish the degree to which there are words that can be recognised, but not defined.

### Assessing Māori vocabulary size

The primary purpose of this paper is to assess the size of the active Māori vocabulary among typical non-Māori-speaking New Zealanders. Assessing vocabulary size has been a productive area of research in applied linguistics over the last 20 years, with a number of reliable measures being devised to ascertain the depth and breadth of vocabulary knowledge in both native and second languages [8,9]. This literature differentiates between different types of ‘knowing’, such as being able to recognise a word, through to being able to select the correct meaning from multichoice options, through to being able to provide a definition for a word [10].

Despite these advances, we have sparse systematic data on how many Māori words the typical non-Māori-speaking New Zealander knows. Deverson [11] estimated that the average non-Māori-speaking New Zealander had a passive knowledge of 40–50 Māori words (excluding place names), a result confirmed by Bellett [12] who tested 143 New Zealanders about whether they knew the meanings of 100 selected Māori words. In the 2000s, Macalister [13] estimated that the average non-Māori-speaking New Zealander knew the meaning of about 70–80 words, based on extrapolations from multichoice questionnaire responses collected from senior secondary school students. No study has estimated how many words New Zealanders can actively provide a definition of (as opposed to selecting the correct definition from a set), nor has any attempt been made to distinguish more passive from more active types of knowledge within the same study. Some insight in these areas can be provided by corpus-based studies of the number and type of Māori words that New Zealanders use in written and spoken language [2,4,14–18]; however, because language usage is affected by a variety of factors, such studies do not definitively address questions of how many Māori words a typical individual actively knows the meaning of.

In this paper, we directly probe Māori word knowledge held by non-Māori-speaking New Zealanders, at two levels: first, at a level of passive or implicit knowledge, in an identification task; and second, at a level of active or explicit knowledge, in a definition task. We use a set of Māori words that we believe are most likely to be known by New Zealanders, which provides a good indication of the size of the typical active Māori vocabulary: if some subset of even these highly recognisable words cannot be reliably defined, then the active vocabulary cannot be large.

### Assessing the role of phonotactics in active Māori lexicon

The secondary purpose of this paper is to compare how different levels of word knowledge modulate effects on the accessing of that knowledge. Do different factors affect how someone responds to a word in an experimental situation when they explicitly know it, as opposed to when they only implicitly recognise it? To answer this question, we look at a factor that is known to affect lexical access: phonotactic probability.

Phonotactic probability is a statistical encoding of gradient well-formedness that captures what words typically ‘sound’ like in a language, based on the extent to which subsequences of phonemes are evidenced across a range of words in that language. A body of work has established that speakers, listeners, and learners of a language have detailed statistical knowledge of phonotactic probabilities, which they can leverage for tasks such as segmenting words from a stream of speech [19–24] and judging the well-formedness of non-words [25–29]. Results from the speech perception literature show that phonotactic probability can affect lexical access [30–33].

**Fig 1:**
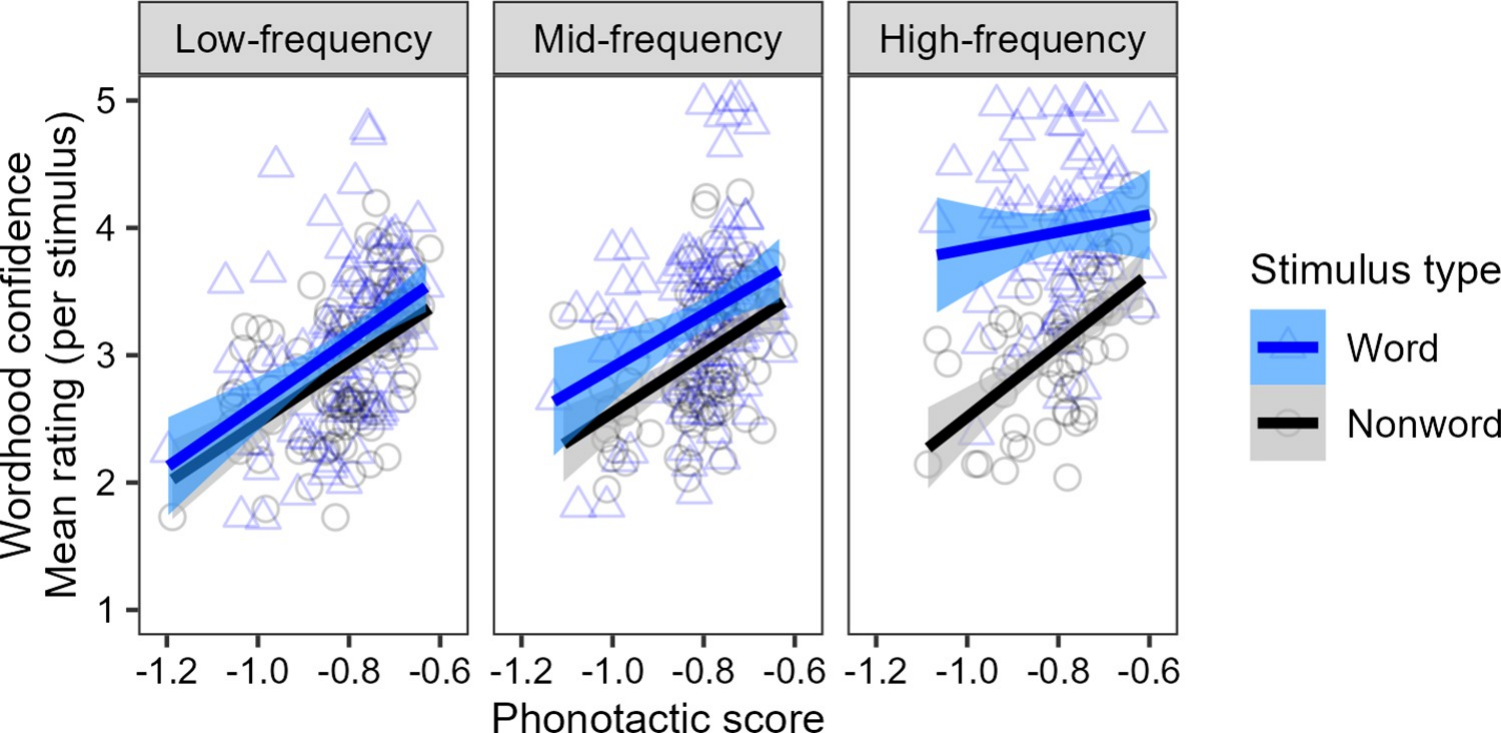
Non-Māori-speaking New Zealanders’ mean wordhood confidence ratings for real word and non-word stimuli per frequency bin, and their relation to phonotactic score. (Licensed under Creative Commons Attribution 4.0. https://creativecommons.org/licenses/by/4.0).

In the context of a proto-lexicon, true lexical access–looking up a form and retrieving its meaning–is not possible as there are no form-meaning pairs. However, there is an analogue in word identification–looking up a form to see if it exists in the memory store. Oh et al. [6] show that phonotactic probability affects this process: non-Māori-speaking New Zealanders rate both words and non-words as more likely to be real Māori words the higher their phonotactic probability. Panther et al. [7], using different stimuli, show that this phonotactic effect levels out for the very highest frequency words, which do not show a phonotactic effect and are reasonably confidently identified (see Fig 1).

Raw data from the Word Identification task in Panther et al. [7] showing mean wordhood confidence ratings for real word (blue triangles) and non-word (black circles) stimuli. Panels show three different frequency bins, in which words of a similar frequency were matched with non-words based on phonotactic score and overall word shape. Separation of words and non-words increases in higher frequency bins. There is a significant effect of phonotactics for all stimuli, with the exception of high frequency real words.

The findings of Panther et al. [7] seem to suggest that Māori-like phonotactics increases ratings of stimuli only for words that participants don’t explicitly know. When participants have explicit knowledge that a stimulus is a word, they respond based on that knowledge, with no further boost from the phonotactics. Indeed, in Oh et al. [6] and Panther et al. [7], we find that although participants are able to distinguish between words and non-words on a rating scale, their confidence is often not high–that is, even though words receive consistently higher ratings than non-words, there is little indication that participants explicitly know the words. The lack of overt, confident, semantic knowledge involved in this task may contribute to the role of phonotactics in influencing the judgements.

Oh et al. [6] and Panther et al. [7] both show an overall frequency effect, with higher frequency words better discriminated from non-words than lower frequency words, and phonotactically well-formed words in general receiving higher ratings. But neither includes words expected to be very highly familiar–that is, words that are sometimes borrowed into English. Would these words, some of which we expect participants to know quite well, reach a ceiling in terms of discriminability? Or would they show some signs of phonotactic influence? We address these open questions in this study by using highly frequent and salient words that we strongly expect participants to know, rather than words of varying frequency as in previous studies [6,7]. Moreover, by using tasks that separately probe implicit knowledge of word form and explicit knowledge of word meaning, we also investigate how the influence of phonotactic probability differs across levels of word knowledge. Does phonotactics directly affect the ability to actively define known words, just as it affects the ability to identify potentially-unknown words, or is the acquisition of semantic knowledge separate from phonotactic probability?

## Aims

The aim of this study is twofold. Primarily, we aim to assess the size of non-Māori-speaking New Zealanders’ active Māori lexicons. Secondarily, we aim to understand the effects of phonotactic cues on these participants’ word judgements, and their ability to associate semantic content with words.

The paper thus has two overarching research questions:

1. How large is the active Māori lexicon of non-Māori-speaking New Zealanders?
2. How does phonotactic knowledge affect the contents of the active lexicon?

Because we explicitly want to distinguish word-form knowledge from the semantic ability to actually define a word, we developed a two-stage approach to trying to determine the approximate vocabulary size of non-Māori-speaking New Zealanders. First, we attempted to find word-forms that they could accurately identify as being words of Māori. Then, we probed their semantic knowledge by asking them to provide definitions for a list of common Māori words. The study thus consists of two online experiments with non-Māori-speaking New Zealanders.

In Experiment 1, we conduct online identification tasks asking non-Māori-speaking New Zealanders to rate their level of confidence that each stimulus is an actual Māori word or phrase (composed of two words) using a 1-to-5 scale. The list of stimuli is comprised of Māori words and Māori-like non-words (containing high-probability phonotactic sequences in Māori).

After determining the list of Māori words identified by most of our participants in Experiment 1, we conduct Experiment 2, an online Māori word definition task. A primary goal of Experiment 2 is to assess non-Māori-speaking New Zealanders’ semantic knowledge of Māori. For both studies, we also assess whether phonotactic probability influences their ability to identify Māori words (Experiment 1) and associate meanings with them (Experiment 2).

## General methods

### Participants

Participants were recruited online via Facebook and Twitter through our social networks. They needed to be adults (18 years or older), who do not speak Māori. They needed to have grown up speaking English in New Zealand, or have lived in New Zealand for at least two years, and not be speakers of te reo Māori.

We removed 29 participants with non-qualifying demographics or outlying response patterns, as outlined in the supplementary materials. After removing outliers, there are 101 and 123 non-Māori-speaking New Zealander participants in Experiments 1 and 2 respectively. Those who completed Experiment 1 are not prohibited from participating in Experiment 2, which was conducted one month after finalising Experiment 1.

To detect any suspicious or unusable participants in Experiment 2 (Māori word definition task), every participant’s definitions of the five words rated as most familiar in Experiment 1 (*haere mai* ‘welcome’; *kia ora* ‘hello’; *haka* ‘traditional war dance’; *kai* ‘food’; *kapa haka* ‘Māori performing arts’) were verified. There were no suspicious participants detected based on this criterion.

Following both experiments, a background information questionnaire was used (see Technical Supplement 3.1 for the questions on the post-questionnaire in S1 File). This questionnaire included questions about demographics, location, and linguistic background, as well as self-reported ability to speak and comprehend Māori (separately), knowledge of basic vocabulary items, and degree of exposure to Māori. The demographic profile of the final set of participants is available in the supplementary materials.

All research protocols were approved by the Human Ethics Committee at the University of Canterbury (Approval number: 2017–90) and the participants provided their written (online) consent to participate in this study.

### Predictor variables

#### Phonotactic scores

Phonotactic score is our representation of phonotactic probability, normalised for stimulus length. We calculated phonotactic score using a triphone-based phonotactic probability that was identified by Oh et al. [6] as most closely explaining non-Māori-speaking New Zealanders’ well-formedness ratings of non-words. It ignores vowel length distinctions (signalled orthographically by a macron) and is based on the 1,629 most frequent morph types derived from all words in the Te Aka dictionary [34], assuming that participants are attempting to parse stimuli into morphs. The use of triphones allows the model to take specific positional information within words into account (i.e. word-initial, word-medial, and word-final syllables), as well as local harmony and disharmony. Following Oh et al. [6], the list of Māori-like non-words is created by using training data composed of two large running speech corpora [35,36] and a dictionary [34]. Some non-words therefore contain trigrams which are not observed in the trigram language model built from a dictionary. To deal with unknown trigrams in non-words, we use the Witten-Bell smoothing implemented in SRILM [37].

#### Neighbourhood density

The neighbourhood density of a word is another factor which has been assumed to influence early word learning [38]. The neighbourhood density of a word is the number of other words which are phonologically similar to a word in a lexicon, but differ from it by one phoneme [25]. Neighbourhood density is related to phonotactic probability [25,26]. Vitevitch and Luce [26] argue that the effects of phonotactic probability and neighbourhood activation are combined and interact in the process of spoken word recognition.

Oh et al. [6] did not consider neighbourhood density, but Panther et al. [7] show an effect of neighbourhood density in the discrimination task, with stimuli with higher neighbourhoods receiving generally higher ratings. This did not interact with frequency or word type. We thus do not necessarily expect any interactions in this study, but include it as a control. Neighbourhood density is the number of words (from the Māori dictionary [34]) that can be reached by adding, deleting, or substituting one phoneme in each stimulus.

#### Macron and length

We include the presence of a macron in our models. While Oh et al. [6] did not test for the effect of a macron in this task, their non-word rating task did show an effect of macrons, with words containing macrons apparently appearing more Māori-like. Panther et al. [7] explicitly avoided macrons in their stimuli. We are unable to avoid them due to the focus on a particular vocabulary subset, so include this as a control. As our stimuli are of variable length, we also include length in phonemes as a control variable. For stimuli that consist of two words, we ignore the space in calculations of phonotactic score, neighbourhood density, and phonemic length, effectively treating each two-word phrase as a single long word.

## Experiment 1: Identification of Māori wordforms

### Stimulus preparation

The stimulus materials for the first experiment, which tests identification of Māori wordforms, comprise of a list of 132 Māori words frequently used in English (including nine phrases consisting of two words) and 209 Māori-like nonwords (including 18 phrases consisting of two Māori-like nonwords). (See Technical Supplement 3.2 for the list of stimuli in S1 File).

The Māori words and phrases were selected impressionistically as being some of those which might be most familiar to participants. For example, we included the words for the numbers 1–10, basic colour words and some commonly used words for body parts. Also included were common greetings like *kia ora* and verbs such as *haere* (‘to go’) and *ako* (‘to learn’). The non-words were generated using a pseudoword generator [39].

About 10,000 non-words (1,000 non-words for each phoneme length of words ranging from length 3 to length 12) were generated by using a Māori dictionary [34] and two running speech corpora [35,36] as training data for the non-words. In order to select filler items for the identification task, words and non-words are classified into different categories based on their phoneme length and the type of their first segment (consonant or vowel) along with their *phonotactic score*. We designed the experiment such that there would be more nonwords than real words in the stimulus set (so as to make the experiment challenging), but wished to avoid making the experiment too long for participants. Thus, for each stimulus category of real words (of size N), we selected 1.5*N non-words. Within each category, we selected non-words with the highest phonotactic scores, so as to make the non-words represent plausible foils for Māori lexical items.

In the experiment, all participants are presented with the same set of 341 stimuli, each time in a different random order. They are provided with an instruction that the list of stimuli contains some Māori words (or phrases) and some Māori-like non-words (or phrases). Stimuli are presented orthographically, rather than auditorily, in order to remove the influence of phonetic factors such as how confident the speaker sounds. When participants are able to map between orthographic and phonological forms, written stimuli can activate phonological knowledge above and beyond orthographic knowledge [40]. We assume this to be the case here because of the transparent nature of the Māori spelling system and the high degree of knowledge that New Zealanders have of it, as described in the Background section.

### Procedure

In Experiment 1, participants see a stimulus in the middle of the screen, which can be either a real word (or real two-word phrase), or a Māori-like non-word (or a phrase of two non-words). Participants are asked to rate how confident they are that each item is an actual Māori word or phrase, using a five-point rating scale ranging from 1 to 5. To clarify the rating scale, the following additional descriptions were added next to the endpoints of the scale respectively–the lowest rating ‘1’ means that participants are ‘Confident that it is NOT a Māori word’ and the highest rating ‘5’ indicates that participants are ‘Confident that it IS a Māori word’. After finishing the rating task, participants are asked to complete a post-questionnaire which includes 19 demographic questions.

### Overall results

The results of the Māori word identification task reveal that, consistent with Oh et al. [6] non-Māori-speaking New Zealanders can discriminate between real Māori words and Māori-like non-words and are able to recognise most of our target stimuli as Māori words.

**Fig 2:**
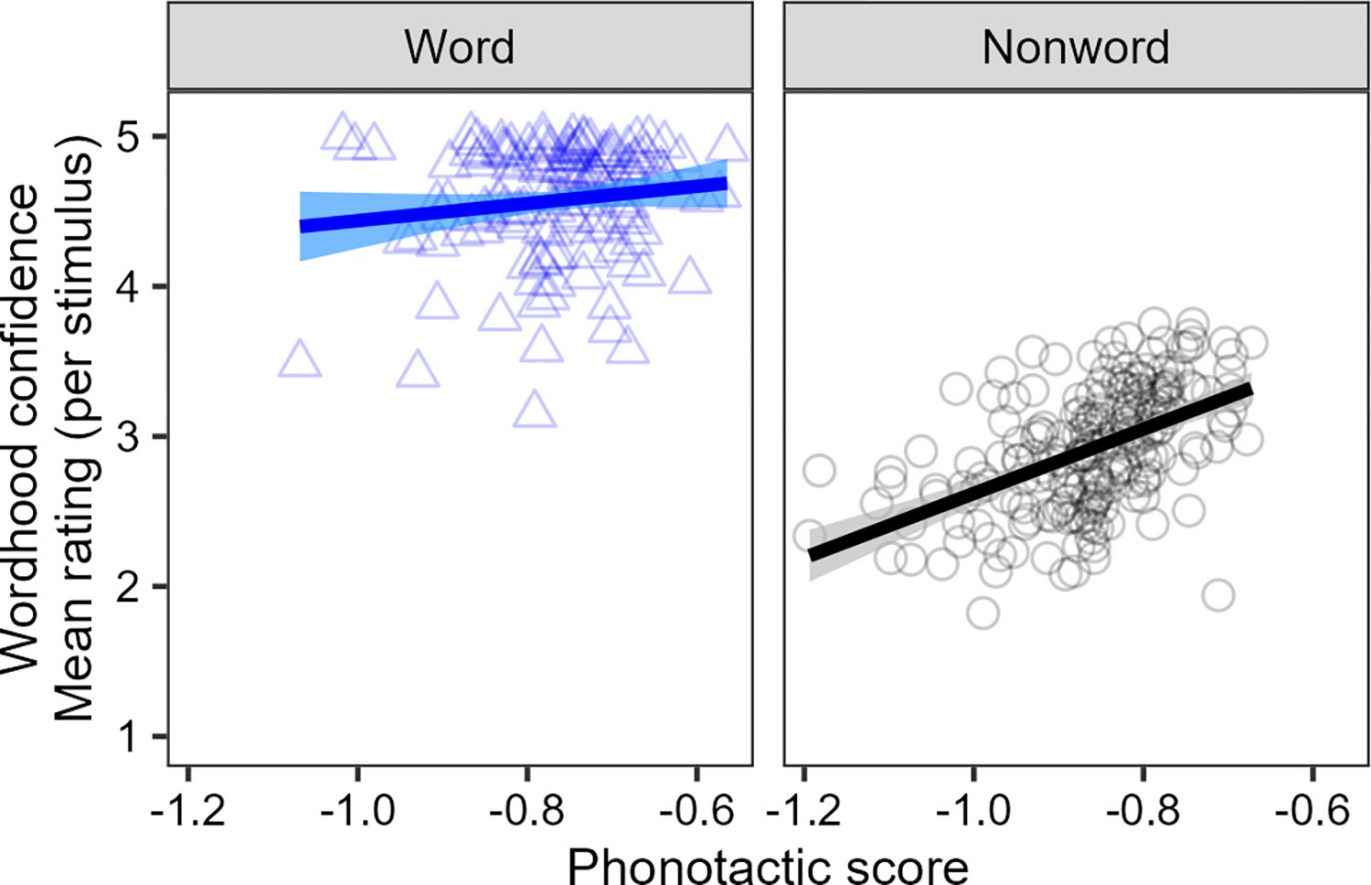
Raw data from Experiment 1 (Word Identification task) showing mean wordhood confidence ratings for real word (blue triangles) and non word (black circles) stimuli.

In Fig 2, the average ratings for 132 words are displayed in the left panel (4.6 on average among real words) and the average ratings for 209 nonwords are shown in the right panel (2.88 on average among nonwords). Among 132 words, only the following 11 words are rated below 4 on average (*ako*, *hope*, *ihu*, *kaiwhakahaere*, *karu*, *mate*, *pango*, *pokohiwi*, *turituri*, *upoko*, *waha*) and the rest of them are rated above 4. The word with the lowest average rating (3.25) is *hope* (‘waist’) which also exists as a word in English.

We fit a (logit) ordinal mixed-effects regression model, considering the predictors of interest–word type (word or non-word, treatment coded with non-word as the reference level) and phonotactic score–and including control factors of number of neighbours, stimulus length and the presence of macron (treatment coded with absent as the reference level). The model is fit in a stepwise procedure, described in detail in the supplementary materials. The initial fitting procedure focuses on fixed effects only, and later stages add intercepts and slopes. The process considers all two-way interactions between a predictor of interest and a control predictor, as well as the interaction between the two primary predictors of interest.

The summary of the selected ordinal mixed-effects regression model is given in Table 1. We see main effects of the number of neighbours and the presence of a macron. Consistent with the result reported in Panther et al. [7], an increased number of neighbours increases the predicted rating, for both words and non-words. Consistent with the result reported in the well-formedness rating task of Oh et al. [6], the presence of a macron in the stimuli also increases overall ratings. Factors that influence word identification ratings in lower frequency words in previous studies, then, also influence word identification ratings in this task involving considerably more frequent words. The neighbourhood density effect suggests that participants are relying on proto-lexical word-knowledge, supporting the conclusion of Oh et al. [6] that they have a proto-lexicon, and suggesting that they can leverage this knowledge to do more than just judge phonotactic probability.

**Table 1:** Model summary for Experiment 1.

	Parameter	Estimate	Std. Error	*z*	*p*	
**Effects**	Phonotactic score (centered)	3.974	0.946	4.201	<0.001	***
	Type = real word	4.734	0.241	19.608	<0.001	***
	Macron = TRUE	0.700	0.180	3.891	<0.001	***
	Neighbourhood density (centered)	0.025	0.011	2.222	0.026	*
	Phonotactic score (centered) × Type = real word	-3.212	1.577	-2.037	0.042	*
**Thresholds**	1|2	-3.073	0.168			
	2|3	-0.775	0.166			
	3|4	1.479	0.166			
	4|5	2.959	0.167			

Model is a mixed-effects (logit) ordinal regression model, with the maximal random effect structure that would converge.

Finally, the model reveals a significant interaction between the phonotactic score and word versus non-word stimuli, as suggested by Fig 2. For non-words, we see an effect of phonotactic score, with more phonotactically probable non-words receiving higher ratings. Conversely, for words, there is little effect of phonotactic score on ratings.

We also note that the rating of the words sits above 4 on average, and at or close to 5 for many words–that is, participants have a high level of confidence that these are real words. This contrasts with the highest frequency bin for Panther et al. [7], where the mean rating is at or slightly below 4, and the separation between words and non-words at high phonotactic scores is not so robust (cf. Fig 1). With this set of high frequency words, participants remain uncertain about the non-words, and are thus influenced by phonotactics, but are more confident in the real words regardless of phonotactic probability.

#### Investigation of individual differences

Given that phonotactic sensitivity appears to not be relevant for words that are well-known, we asked whether the patterns we see hold true over the whole data set, or whether they are different for participants with different degrees of apparent knowledge of Māori.

To measure a participant’s apparent knowledge of Māori, we use d-prime [41] which measures their sensitivity to real words vs. non-words in the experiment (see supplementary materials for details of implementation). Intuitively, d-prime can be regarded as a measure of *how much* Māori word knowledge participants have, with participants with higher values of d-prime having more knowledge. In this analysis, we are interested in whether participants with different degrees of word knowledge show different degrees of sensitivity to phonotactic score, presence of a macron, and number of neighbours. Thus, we build on the previous ordinal regression model to allow every term to interact with d-prime, and we investigate the nature of these interactions. The resulting model is shown in Table 2. It shows significant interactions between d-prime and each of our previous predictors.

**Table 2:** Model summary for investigation of individual differences in Experiment 1, using participant d-prime.

	Parameter	Estimate	Std. Error	*z*	*p*	
**Effects**	D-prime (centered)	-0.321	0.175	-1.832	0.067	.
	Phonotactic score (centered)	4.039	0.950	4.252	<0.001	***
	Type = Real word	4.625	0.196	23.621	<0.001	***
	Macron = TRUE	0.693	0.176	3.932	<0.001	***
	Neighbourhood density (centered)	0.025	0.011	2.234	0.025	*
	D-prime (centered) × Phonotactic score (centered)	-0.755	0.348	-2.169	0.030	*
	D-prime (centered) × Type = Real word	1.943	0.153	12.735	<0.001	***
	D-prime (centered) × Macron = TRUE	-0.586	0.107	-5.468	<0.001	***
	D-prime (centered) × Neighbourhood density (centered)	0.024	0.006	4.026	<0.001	***
	Phonotactic score (centered) × Type = Real word	-3.174	1.583	-2.005	0.045	*
**Thresholds**	1|2	-3.058	0.157			
	2|3	-0.725	0.155			
	3|4	1.464	0.155			
	4|5	2.937	0.156			

Model is a mixed-effects (logit) ordinal regression model, with the maximal random effect structure that would converge.

**Fig 3:**
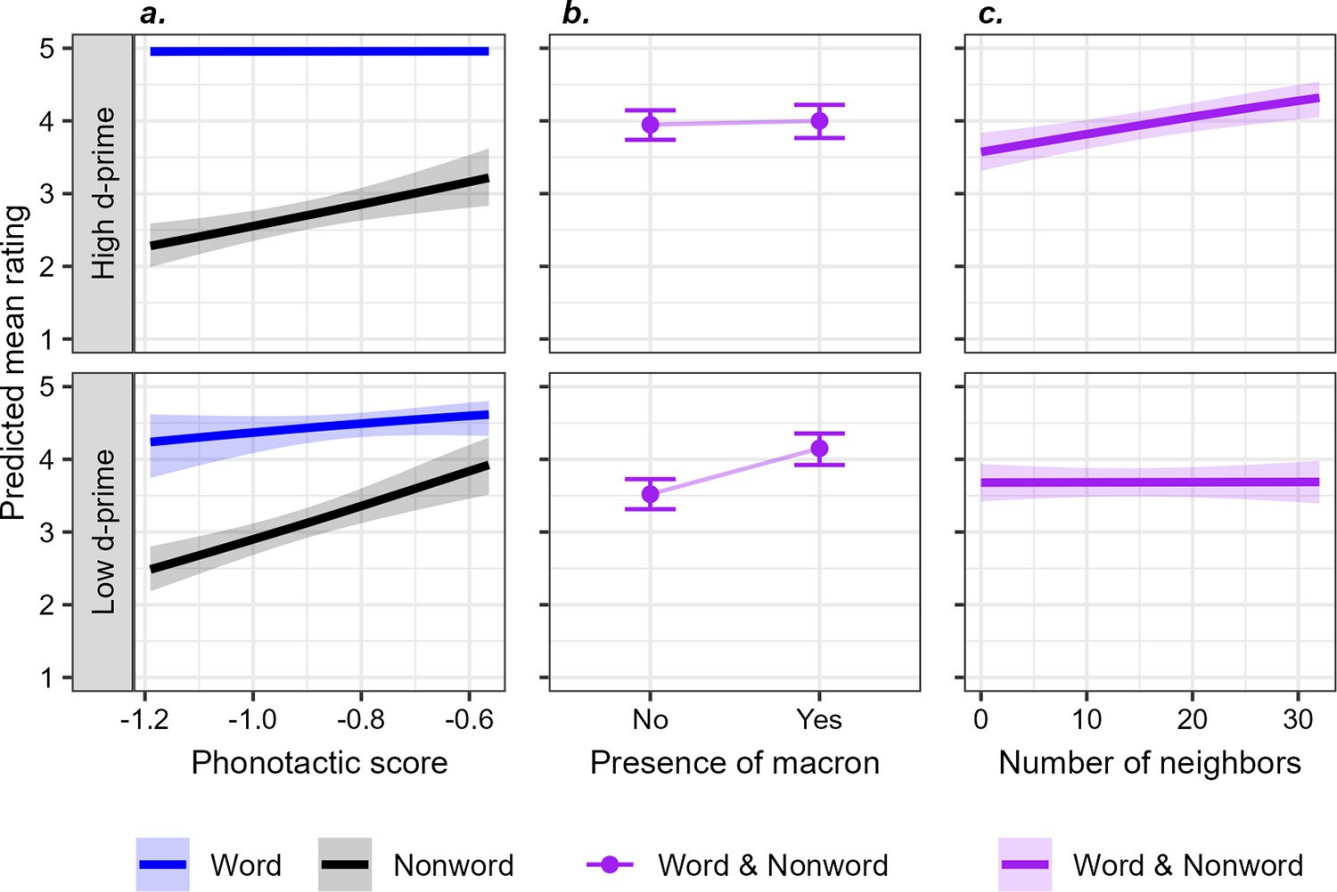
Partial effect plots for predicted ratings. It shows interactions of participant d-prime with effects of: (a) the interaction of phonotactic score and stimulus type (word or non-word); (b) the presence of a macron; (c) the number of phonological neighbours. Participant d-prime was entered in the statistical model as a continuous variable, but is taken at two fixed values here for illustrative purposes: high (d-prime = 3) and low (d-prime = 1).

The partial effect plots for the interactions are given in Fig 3. As expected from the definition of d-prime, participants with a lot of Māori word knowledge (high d-prime) show a higher degree of separation between ratings for words and non-words than participants with less Māori word knowledge (note that d-prime was modeled as a continuous variable, and has been broken into dichotomous values here for illustrative purposes only). But beyond this simple effect, there are many differences in degree of sensitivity to other factors as well: as a participant’s word knowledge increases, they make less use of phonotactic score in rating non-words (Fig 3A), less use of the presence of macrons (Fig 3B), and more use of phonological neighbours (Fig 3C). Taken together, this suggests that a higher degree of word knowledge allows a participant to use more *specific* and *explicit* knowledge in this task: rather than using presence of a macron as a proxy, or a sort of generalised “Māori-like-ness” offered by phonotactic score, they make their ratings based on confident awareness of the existence of a word where possible, and based in part on awareness of specific highly-similar words (phonological neighbours).

### Experiment 1: Summary

Experiment 1 has shown that non-Māori-speaking New Zealanders were, on the whole, very accurate at discriminating the real Māori words in our experiment from the non-real foils. There were 121 words that were identified, on average, with a high degree of confidence (average rating >4). Significant effects of phonotactic and neighbourhood cues on non-Māori-speaking New Zealanders’ ratings also reveal that they possess sophisticated proto-lexical knowledge which makes them more likely to accept highly Māori-like non-words than less Māori-like non-words. Moreover, increased word-knowledge led to changes in the use of other linguistic cues for this task, in two ways. First, for the set of real words in this experiment, which are in general very familiar, we do not see any phonotactic effect. And second, when we look across individual participants, those with greater discrimination between words and non-words are relying less on the visual cue of the macron, and more on lexical neighbourhood effects–which require some degree of lexical or proto-lexical knowledge.

Given the results from Oh et al. [6] and Panther et al. [7], it should not be surprising that there is a high degree of discrimination between words and non-words in this task. An important question, addressed in the next experiment, is the degree to which the words that were well recognised can also be defined.

### Experiment 2: Definition of Māori words

#### Stimulus preparation

Among 132 words tested in Experiment 1, there were 11 words which scored below 4 on average and these were discarded for Experiment 2. The words *Aotearoa*, *Māori*, and *Pākehā* were also removed, because providing definitions for these highly-frequent nouns may not be trivial–there are no straightforward English translation equivalents. For the second experiment, which tests participants’ active lexical knowledge of Māori, the list of stimuli therefore consists of 118 words (See Technical Supplement 3.2.2 for the list of stimuli in S1 File).

#### Procedure

Before starting the definition task, participants are provided with an instruction to give their best guess without looking up words in a dictionary or asking for others’ help. Furthermore, we explain to the participants that we are interested in gauging each individual’s actual knowledge of Māori words’ meanings, more so than collecting correct definitions. During the definition task, participants are asked to type a definition of a Māori word they see in the middle of the screen and type ‘NA’ if they do not know the meaning of the word. However, in order to encourage them to give their best answer without marking a lot of NAs, we add an additional question below on the same screen, which asks them to rate how confident they are with their definition. The scale of confidence rating ranges from 1 (‘Not confident’) to 5 (‘Very confident’). During the first part, the identical set of 118 stimuli are presented in a different random order to each participant. After finishing the definition task, participants complete a post-questionnaire which is the same as the one used in Experiment 1. The accuracy of the definitions in Experiment 2 were hand-coded by a fluent speaker of Māori.

#### Results

**Fig 4:**
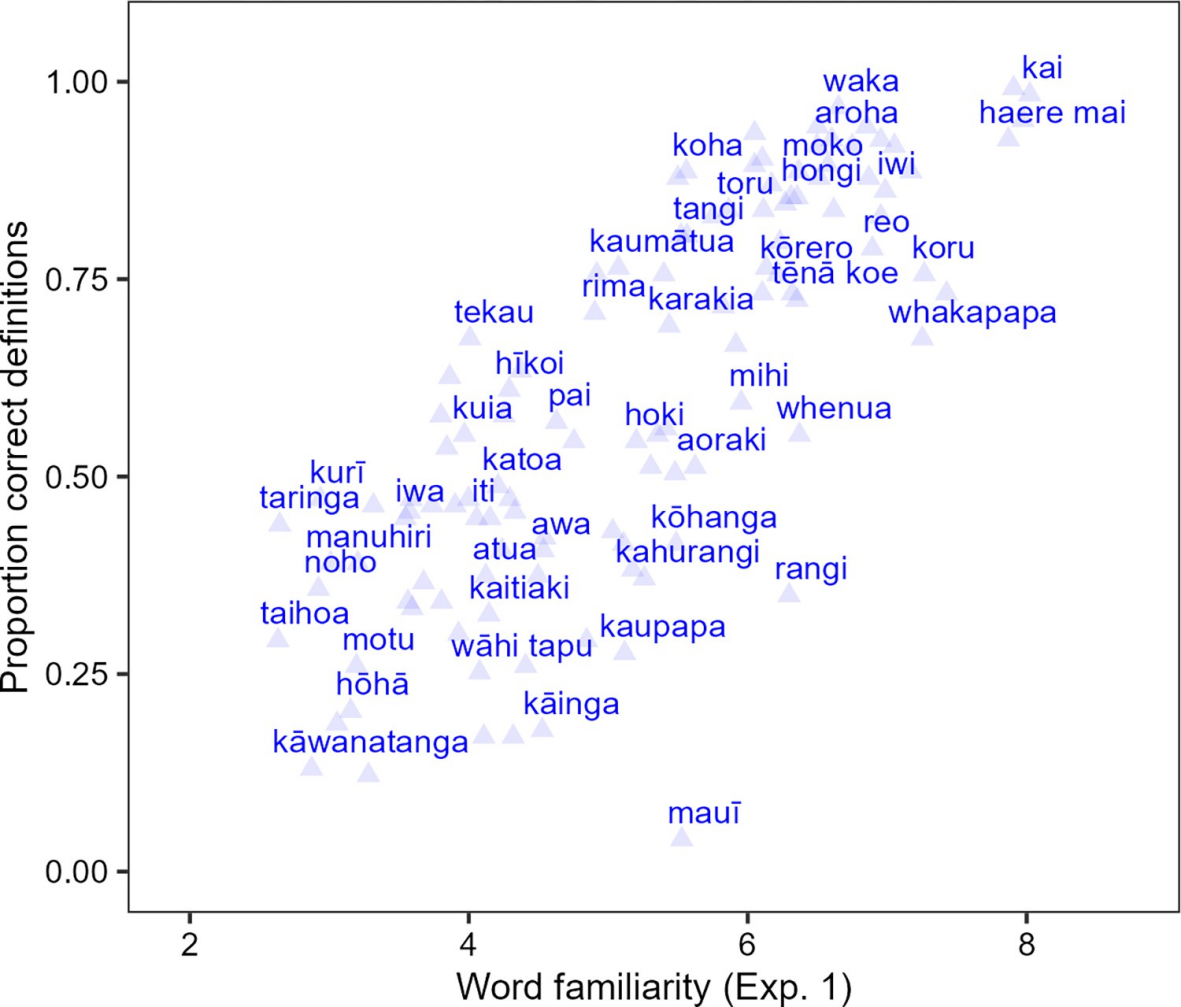
Proportion of correct definitions as a function of familiarity, based on the predicted mean ratings from Experiment 1. Selected words are labeled adjacent to the corresponding point.

For each word, the rate of getting correct responses differs substantially, ranging from 4% for *mauī* (‘left’) and 99% for *kia ora* (‘hello’). Fig 4 displays the proportion of correct responses per word in the definition task performed by 123 non-Māori-speaking New Zealanders. The extent to which a word is able to be correctly defined is highly correlated with its mean rating from Experiment 1. The participants provided correct definitions with more than 50% chance for 70 words out of 118 words (59%), which implies that for a significant subset of the words they can identify as Māori words in Experiment 1, they are not able to provide accurate definitions of those words.

The range across respondents was large–ranging from 8 to 116 correct items, with a mean of 71, a median of 72, and a standard deviation 24. Viewed by participant, then, we also reach the conclusion that the average New Zealander can define about 70 words, although there is considerable variability across participants. This, then, provides an answer to our first research question, regarding an approximate estimation of the active Māori vocabulary of New Zealanders.

To assess the extent to which various factors affected participants’ ability to accurately define words, we use mixed-effects logistic regression. The dependent variable in each model is the accuracy of response; this is a categorical outcome variable indicating whether or not the participant was able to provide an accurate definition for the word / phrase. In particular, we are interested to know if the factors that affected awareness / recognition of words in Experiment 1 –phonotactic score, presence of a macron, and number of phonological neighbours–offer additional advantages in being able to *define* words. We included all the fixed effects from Experiment 1 (with the exception of type, as all words in Experiment 2 are real words), and added a predictor of familiarity–the predicted mean rating for the word from Experiment 1, added in a manner that controls for nonlinearities and clustering (see Technical Supplement 2.5 in S1 File).

We observed in Experiment 1 that participants’ sensitivities to word properties differed with their degree of apparent Māori word knowledge. In a similar way, it is possible that participants’ sensitivity to word properties in being able to define words accurately in Experiment 2 will differ based on how widely known the word is. For this reason, we include interactions between familiarity and the predictors that showed an effect in Experiment 1 – phonotactic score, macron, and neighbours. We do not include additional interactions, since we have no theoretical reason to suspect them, and since the number of words for which we have data is relatively small.

We again take a stepwise approach to model fitting, as described in detail in the supplementary materials. This involved the addition of random slopes into the model after an initial procedure involving intercepts only.

As shown in Table 3, the only significant predictor was the familiarity from Experiment 1, which can be seen in the close relationship between familiarity and definition accuracy in the raw data shown in Fig 4. Words that received high ratings in Experiment 1 were more accurately defined in Experiment 2. The linguistic features had no separate effect on definition accuracy.

**Table 3:** Model results for Experiment 2 (modelling definition accuracy).

Parameter	Estimate	Std. Error	*z*	*p*	
(Intercept)	0.773	0.180	4.300	<0.001	***
Familiarity.latent (centered)	1.068	0.083	12.837	<0.001	***
Phonotactic score (centered)	0.080	1.406	0.057	0.955	
Macron = TRUE	-0.502	0.279	-1.799	0.072	.
Neighbourhood density (centered)	-0.029	0.020	-1.413	0.158	
Length (centered)	-0.106	0.079	-1.342	0.179	

Model is a mixed-effects binary logistic regression model, with the maximal random effect structure that would converge.

There are two primary conclusions to be drawn from this simple model. First, New Zealanders’ active Māori vocabulary–i.e. the set of Māori words they can accurately define–is small, just as it was estimated to be through different means and at earlier times in previous literature. Our result of 70 words with a definition accuracy of above 50% is relatively consistent with Macalister’s estimate of 70–80 words [13]. We note that our threshold of ‘knowing’ is higher than in Macalister [13]–requiring active definition rather than success in a multi-choice task. We also note that our experiment contains proportionally many fewer terms for flora and fauna than Macalister’s experiments [13]. Thus, although we reached the same number, this result probably does reflect some increased knowledge of core vocabulary items since that time. It is of course also possible there are some words that are widely known that were omitted from our stimulus list, which largely comprised a collective educated guess of which words New Zealanders were likely to know. Second, while linguistic factors such as phonotactics and neighbourhood density play an important role in the degree to which word-forms are recognised, they play no additional role in constraining the learnability of word meanings themselves.

## Discussion

The questions we set out to answer were:

1. How large is the active Māori lexicon of non-Māori-speaking New Zealanders?
2. How does phonotactic knowledge affect the contents of the active lexicon?

We address these questions in the sections below.

### Active Māori lexicon size

With respect to (1), we have shown that the semantic lexicon is much smaller than the number of word-forms that can be passively identified as being Māori words. While results from participants varied, there were 70 words in our experiment that could be accurately defined by more than half of our participants. This confirms previous reports that the active lexicon is not large and does so using a methodology that explicitly probes the ability to actively produce a correct definition. However, the number of words accurately defined is significantly smaller than the number of words which can be recognised as Māori word-forms. This highlights the importance of the task used, when assessing the size of the vocabulary. Assessing changes in the Māori vocabulary size over the last few decades is currently difficult, due to the different methodologies used, all of which have tapped into different degrees of relatively passive knowledge.

This result provides strong empirical support to the claims by Oh et al. [6] and Panther et al. [7] that the impressive word knowledge demonstrated in their experiments does indeed seem to be ‘proto-lexical’ and not stemming from any active knowledge. The participants in this experiment could only actively define 70 of the 132 words we explicitly chose to be most likely recognised by New Zealanders. We can therefore provide an empirical basis for the claim that they would not know the definition of many words in the previous experiments, which were substantially lower frequency, nor the ~1500 words and word parts that appear to be needed to model their phonotactic knowledge (cf. Oh et al. [6]).

### The role of phonotactics in acquiring high frequency words

In terms of our second research question, regarding phonotactics, our results in the discrimination task reinforce the conclusions of Panther et al. [7], that phonotactics affects the general sense of Māori word-likeness in a discrimination task, but when there is overt and explicit knowledge, this phonotactic effect is no longer there. Thus, in this task with high familiarity words, we see an effect of phonotactic knowledge on the non-words, but not the real words–very similar to the highest frequency words investigated by Panther et al [7].

Further evidence that explicit knowledge can reduce reliance on phonotactics comes from the analysis of individual variation, which shows that the effects of phonotactics and of having a macron also lessen in participants with high levels of overt knowledge. Conversely, these participants presumably have stronger lexical knowledge, and thus the neighbourhood density effect strengthens.

The interpretation that factors such as phonotactics can become less important with overt knowledge also supports the results of the definition task, where we find no significant predictors except for accuracy in the discrimination task. Words that were more accurately identified as real in the discrimination task were also better defined. There was no further effect of phonotactics, macron, or neighbourhood density. Participants either know the word, and can define it, or they do not. These linguistic factors appear to have had no additional direct role in the likelihood of acquiring the word meaning once a word becomes readily recognisable; however, such factors do still play an indirect role, in determining the recognisability of a word in the first place.

We know that phonotactic effects have been found in classic results on the effect of phonotactic probability in lexical decision in English (e.g. [26]). Analysis in that literature is primarily of reaction times, although the above-mentioned work also looks at accuracy, finding more accurate lexical decision for stimuli with low phonotactic probability than for stimuli with high phonotactic probability.

In this context, it may seem superficially surprising that our discrimination task does not show any strong effect of phonotactics for real words. However a crucial difference between our task and a classical lexical decision task is that our task is not speeded. Errors in lexical decision tasks with native speakers come from errors induced by speed, rather than from absence of relevant lexical knowledge, whereas errors in our unspeeded task come from not recognising the word. Indeed, Vitevitch and Luce assume that the speeded nature of the task as key to interpreting their results: “a response may be initiated when activation for a unique lexical item has reached some criterion or threshold. However, when a single lexical item fails to receive sufficient activation within the time period required for a response, decisions may be based on the overall level of lexically based activity in the recognition system [26] (p.387)”. In our task, there is always time for a representation for a lexical item (if it exists) to reach threshold. Errors for our real words are driven by actual absence of lexical knowledge.

Our task also differs from classic lexical decision in other ways, including the use of a rating scale, making a direct comparison between our results and work on English impossible. What our results do highlight is that more work is required to fully disentangle the role of phonotactic knowledge across different tasks and across different linguistic contexts.

### Supporting the proto-lexicon

Finally, these results support the conclusions of Oh et al. [6] and Panther et al. [7] that non-Māori-speaking New Zealanders have substantial subconscious knowledge of Māori that they have acquired through ambient exposure. Our results provide empirical support for the claim that this knowledge is not overt–since there are only a small number of Māori words that New Zealanders can actually define. They also provide support in that we replicate in this population the overall sensitivity to properties of the Māori lexicon. Non-word ratings are strongly affected by the phonotactic probability of the non-word. Based solely on the results of this paper, this would seem to set up a paradox–if non-Māori-speaking New Zealanders have such a small explicit vocabulary of Māori, where does their phonotactic knowledge come from? But the answer is provided by the experiments and modelling of Oh et al. [6] who show that there is substantial implicit vocabulary knowledge–without semantics–over which phonotactic generalisations can be drawn. The sensitivity of the participants in our experiments to neighbourhood density, despite their very small active vocabulary, provides further indication that their implicit Māori language knowledge is non-trivial, and can generate neighbourhood density effects. Speakers who have semantic lexicons of fewer than 100 words can show high levels of sensitivity to the statistical patterns in a language.

## Conclusion

We have conducted experiments on New Zealanders who do not speak Māori, in order to assess the size of their active lexicon of the Māori Language. We identify 70 words that can be defined at above-average rates by this population.

This result plays an important role in providing an empirical basis for claims in the wider literature that non-Māori speakers in New Zealand possess a large ‘proto-lexicon’ of words and word-parts that they cannot define and are not actively aware of. We have confirmed that, despite results showing that this population seems to ‘know’ a very large number of words, the size of their active lexicon–words that can be actively defined–is very limited. The contribution of linguistic factors that seem to require a lexicon, such as phonotactics and neighbourhood density, support the claims that the implicit knowledge is much more extensive than this small active vocabulary would suggest.

Phonotactic well-formedness affects the likelihood that a non-word will be mistakenly recognised as a word, but not the success rate for high-frequency real words, nor the likelihood of successful definition of these words. Indeed, a variety of results point to such linguistic factors playing a role in tasks and participants involving low certainty, but not in tasks for which participants can call on active and explicit knowledge.

In general, these results confirm the need for precision in our definitions when investigating vocabulary size, and the factors that may influence this. Many scholars have highlighted the fact that what can be described as “word knowledge” exists on a continuum [42–44]. For Dale [43], for example, the continuum starts at no knowledge–we do not know that the word exists, through awareness without definition, to a vague notion of the meaning, to a rich semantic knowledge. New Zealanders have few words with semantic knowledge, but a large set of words at the very earliest stages of knowledge–earlier, even, than overt awareness of knowing. Precision of definition when talking about word knowledge, then, is vital. In addition, linguistic knowledge can affect tasks tapping one form of knowing but not another. This suggests that investigation of the differential role of linguistic constraints on different types of word knowledge is likely to prove a useful future direction of inquiry.

## Supporting information

S1 File Assessing the size of non-Māori-speakers’ active Māori lexicon. (HTML)
