# Supplementary material for: Assessing the size of non-Māori-speakers’ active Māori lexicon
Source: PLoS One. 2023 Aug 23;18(8):e0289669. doi: 10.1371/journal.pone.0289669 (PMC10446205; doi:10.1371/journal.pone.0289669)
Supplement: S1 File — (HTML) [file pone.0289669.s001.html]

Assessing the size of non-Māori-speakers’ active Māori lexicon


Code 

- Show All Code
- Hide All Code

# Assessing the size of non-Māori-speakers’ active Māori lexicon

### Supplementary materials

#### 2022-11-04 15:38:28

# 0 Introduction

This R Markdown document contains all the code used for outlier detection, data analysis, and plotting, including all the statistical models with summaries. It also lists the questions from the post-experiment questionnaire and the stimuli used in both experiments.

# 1 Experiment 1

Oh et al. (2020) found that non-Māori-speaking New Zealanders were able to distinguish real Māori words from Māori-like nonwords, and that their ratings in this task were affected by the phonotactic probability of a stimulus (word or nonword): the higher the phonotactic probability of the stimulus, the more confident participants were that it was a real Māori word. These effects held for words of all frequencies. Panther et al. (under review) replicated these effects, and their raw data suggested that the effect of phonotactic probability may be weaker for high-frequency words (which participants are likely to have encountered often and thus may know or recognize explicitly) than for low-frequency words (which participants are less likely to know explicitly). In Experiment 1, we investigate this idea, by asking whether the effects of phonotactic probability can be replicated among words of which participants are likely to have explicit awareness and recognition. Experiment 1 also serves to establish which words participants are, indeed, readily able to recognize, to feed into our investigation of their knowledge of those words in Experiment 2.

In Experiment 1, participants were presented with a series of word and non-word stimuli, one at a time, and rated how confident they were that each was a real word of Māori. The real words were carefully hand-selected based on the intuition that many non-Māori-speaking New Zealanders are assumed to be exposed to them and to have some degree of knowledge of them; this intuition stems from their frequency of use in Māori and in New Zealand English (as borrowings). Due to these selection criteria, we expected non-Māori-speaking New Zealanders to be able to recognize many of the words in the experiment. The non-words were constructed by concatenating valid Māori syllables in such a way as to span a similar range of lengths as the real words, and were then filtered to remove items with low phonotactic probability.

## 1.1 Data

### 1.1.1 Exclusions

For Experiment 1, we excluded:

- 11 participants with suspicious patterns of responses, suggesting they did not complete the experiment in good faith;
- 4 participants with a negative \(d^\prime\), indicating that they rated a higher proportion of nonwords than real words with 4 or 5 (‘confident that is IS a real word’), thus performing worse than chance (which is highly unexpected given the high-profile nature of the real words);
- 1 participant who was not a native speaker of English;
- 11 participants who indicated above-basic ability to speak or comprehend te reo Māori (i.e. rated greater than 2 on a scale of 0-5 for either speaking or comprehension);
- 2 participants who learned their English outside NZ and have been living outside NZ for more than 2 years;
- all responses to the stimulus *whanau*, which was supposed to be *whānau* (with a macron).

The exclusion process for Experiment 1 is detailed in the code below.

```
# Loading the data to filter out participants
dataExp1 <- read.delim("./dataAnonNotFilteredExp1_added.txt", sep ="\t", header = TRUE, encoding="UTF-8")

# Remove 11 suspicious participants
rmParticipant1Exp1 <- c("5970a90e","6e5ccd20","9e75832a","86687167","64afd2cc","429604bc","409f2599","d6390ddc","7ab6a320","bd90455b","39b29c2b")
dataExp1 <- dataExp1[!dataExp1$workerId %in% rmParticipant1Exp1,]

# Calculate dprime
# Get the hit rate: 4 or 5 to real words
dataExp1$accReal <- ifelse(dataExp1$type=="real" & dataExp1$enteredResponse %in% c(4,5),1,0)
hrateT <- aggregate(accReal ~ workerId, sum, data=dataExp1)
hrateT$hit_rate <- round(hrateT$accReal/132,3)

# Manually correct the hit rate of one participant (who did one less item)
#length(which(dataExp1$type=="real" & dataExp1$workerId=="c0999383"))
hrateT[hrateT$workerId=="c0999383",]$hit_rate <- round(hrateT[hrateT$workerId=="c0999383",]$accReal/131,3)

# Get the FA (false alarm) rate: 4-5 to nonwords
dataExp1$accNon <- ifelse(dataExp1$type=="non" & dataExp1$enteredResponse %in% c(4,5),1,0)
farateT <- aggregate(accNon ~ workerId, sum, data=dataExp1)
farateT$fa_rate <- round(farateT$accNon/209,3)

# Manually correct the FA rate of one participant (who did two less items)
#length(which(dataExp1$type=="non" & dataExp1$workerId=="f06d0f73"))
farateT[farateT$workerId=="f06d0f73",]$fa_rate <- round(farateT[farateT$workerId=="f06d0f73",]$accNon/207,3)
dprime <- merge(hrateT, farateT, by="workerId")
dprime$dprime <- round(qnorm(dprime$hit_rate) - qnorm(dprime$fa_rate),3)
dprime <- dprime[c("workerId","dprime")]
drop <- c("accReal","accNon")
dataExp1 <- dataExp1[,!(names(dataExp1) %in% drop)]
dataExp1 <- merge(dataExp1, dprime, by="workerId")
rm(dprime, hrateT, farateT)

# Remove 4 participants whose d-prime value is lower than 0
rmParticipant2Exp1 <- unique(dataExp1[dataExp1$dprime < 0,]$workerId) 
# a29fe31d e1f518e6 efdd3439 f57a3633 
dataExp1 <- dataExp1[!dataExp1$workerId %in% rmParticipant2Exp1,]           

# Remove one native speaker of Mandarin Chinese
dataExp1 <- dataExp1[!dataExp1$firstLang=="Mandarin",]

# Remove 11 participants whose speakMaori or compMaori is equal to or above 3
rmParticipant3Exp1 <- unique(dataExp1[dataExp1$speakMaori >= 3 | dataExp1$compMaori >= 3,]$workerId)
# 007a1752 170ce007 20c10896 3128bb29 66d0a920 75caca3b b00cd565 d3cd7085 de95cdaf eef4d9c0 fab8a51f
dataExp1 <- dataExp1[!dataExp1$workerId %in% rmParticipant3Exp1,]

# Remove one participant who did not learn their English in NZ and have been living overseas for more than two years.
summaryExp1WorkerId <- unique(dataExp1[,c("workerId","firstLangCountry","place","duration")])
EngNotInNZExp1 <- summaryExp1WorkerId[!summaryExp1WorkerId$firstLangCountry=="NZ",]
rmParticipant4Exp1 <- unique(EngNotInNZExp1[EngNotInNZExp1$place=="overseas",]$workerId) 
# 880242c2
dataExp1 <- dataExp1[!dataExp1$workerId %in% rmParticipant4Exp1,]

# Detect participant whose median reactionTime is shorter than 2*SD below the mean of all participants
median_RT <- aggregate(dataExp1$reactionTime, by=list(dataExp1$workerId), median)
names(median_RT) <- c("workerId","median")
cut <- mean(median_RT$median)-2*sd(median_RT$median)
# median_RT[!median_RT$median > cut,]$workerId # None detected!

# Check the total number of usable participants for Exp1
# length(unique(dataExp1$workerId)) # 101

# Add a column to indicate the presence of macrons
dataExp1$macron <- FALSE
dataExp1[grepl("\U0101|\U0113|\U012B|\U014D|\U016B",dataExp1$word),]$macron <- TRUE
dataExp1$macron <- as.factor(dataExp1$macron)

# Remove rows containing a word "whanau"
dataExp1 <- dataExp1[!dataExp1$word=="whanau",]
```

### 1.1.2 Dataset structure

The data is structured as follows:

- *workerId* is the unique ID for each participant.
- *enteredResponse* is the confidence rating for each stimulus.
- *reactionTime* is the reaction time for each rating (seconds).
- *type* is the classification of each stimulus: nonword (‘non’) or word (‘real’).
- *length* is the phoneme length of each stimulus.
- *word* is the stimulus used for the rating.
- *speakMaori* is each participant’s report of how well they can speak Māori (on a scale from 0 to 5).
- *compMaori* is each participant’s report of how well they can understand/read Māori (on a scale from 0 to 5).
- *maoriProf* is the sum of quantified response for speakMaori and compMaori (participant Māori proficiency).
- *age* is the age group for each participant.
- *gender* is the gender reported by each participant.
- *ethnicity* is categorized into binary answers, either Māori (M) or non Māori (non M).
- *education* is each participant’s highest level of education.
- *children* is each participant’s report of whether they have had any children who have attended preschool or primary school in New Zealand in the past five years.
- *maoriList* is each participant’s basic knowledge of Māori (with a scale ranging from 0 to 9).
- *place* is each participant’s current place of living (3 levels: NZ North Island, NZ South Island, or Overseas).
- *duration* is each participant’s time living in their current place (2 levels: long is > 2 years; short is =< 2 years).
- *firstLang* is each participant’s first language.
- *firstLangCountry* is the country where each participant learned their first language.
- *anyOtherLangs* is any other languages each participant reports speaking.
- *hawaii* is the binary response to the question whether participants have lived in Hawaii.
- *anyPolynesian* is the binary response to the question whether participants know any Polynesian such as Hawaiian, Tahitian, Sāmoan, or Tongan.
- *whichPolynesian* is the information regarding participants’ knowledge of Polynesian languages, if they know any.
- *impairments* is the answer to the question whether participants have a history of any speech or language impairments.
- *maoriExpo* is each participant’s level of exposure to Māori (with a scale ranging from 0 to 10).
- *score* is the phonotactic score (i.e. triphone-based phonotactic probability, normalized by phonemic length) that Oh et al. (2020) identified as most closely explaining non-Māori-speaking New Zealanders’ wellformedness ratings of nonwords. It ignores vowel length distinctions (signalled orthographically by a macron) and is based on the 1,629 most frequent morph types derived from all words in the Te Aka dictionary, assuming that participants are attempting to parse stimuli into morphs.
- *macron* indicates whether or not the stimulus includes a macron, which orthgraphically indicates a long vowel. We include the presence of macrons in our analysis because it is visually salient, but is not otherwise reflected in terms such as phonotactic score.
- *n.neighbors* is the the number of words (from the Māori dictionary) that can be reached by adding, deleting, or substituting one phoneme in each stimulus.
- *mean.neighbor.logfreq* is the frequency-weighted phonological neighbourhood density.
- *dprime* is the measure of sensitivity for each participant’s performance, reflecting a normalized difference between a participant’s *hit rate* (proportion of real words given a rating of 4 or 5) and their *false alarm rate* (proportion of nonwords given a rating of 4 or 5).

## 1.2 Participants

After exclusions, we were left with data from 101 participants. A summary of the demographics of the participants is presented below.

```
# 1) Gender
Exp1Gender <- unique(dataExp1[,c("gender","workerId")])
Exp1GenderT <- as.data.frame(table(Exp1Gender$gender));names(Exp1GenderT) <- c("gender","freq")
Fig1Exp1Gender <- ggplot(Exp1Gender, aes(x=gender,color=gender,fill=gender)) + theme_classic() + geom_bar(aes(),size=.1,show.legend=F) + geom_text(data=Exp1GenderT, aes(x=gender,y=freq,label=freq), color="black", vjust=-0.2,size=3)+labs(x="Gender",y="Number of participants") + coord_cartesian(ylim=c(0, 80))

# 2) Education
Exp1Education <- unique(dataExp1[,c("education","workerId")]);Exp1Education$education <- as.factor(Exp1Education$education)
Exp1EducationT <- as.data.frame(table(Exp1Education$education));names(Exp1EducationT) <- c("education","freq")
edu_levels <- c("high","undergraduate","graduate")
Exp1Education$education <- factor(Exp1Education$education, levels = edu_levels)
Exp1EducationT$education <- factor(Exp1EducationT$education, levels = edu_levels)
Fig1Exp1Education <- ggplot(Exp1Education, aes(x=education,color=education,fill=education)) + theme_classic() + geom_bar(aes(),size=.1,show.legend=F) + geom_text(data=Exp1EducationT, aes(x=education,y=freq,label=freq), color="black", vjust=-0.2,size=3) + labs(x="Highest education",y="Number of participants") + coord_cartesian(ylim=c(0, 52))

# 3) Basic knowledge of Māori
Exp1ML <- unique(dataExp1[,c("maoriList","workerId")]);Exp1ML$maoriList <- as.factor(Exp1ML$maoriList)
Exp1MLT <- as.data.frame(table(Exp1ML$maoriList));names(Exp1MLT) <- c("maoriList","freq")
Fig1Exp1ML <- ggplot(Exp1ML, aes(x=maoriList,color=maoriList,fill=maoriList))+theme_classic()+ geom_bar(aes(),size=.1,show.legend=F) + geom_text(data=Exp1MLT, aes(x=maoriList,y=freq,label=freq), color="black", vjust=-0.2,size=3) + labs(x="Basic knowledge of Māori",y="Number of participants") + coord_cartesian(ylim=c(0, 20))
  
# 4) Age
Exp1Age <- unique(dataExp1[,c("age","workerId")]);Exp1Age$age <- as.factor(Exp1Age$age)
Exp1Age$age <- factor(Exp1Age$age, levels=c("18-29", "30-39", "40-49", "50-59", "+60"))
Exp1AgeT <- as.data.frame(table(Exp1Age$age));names(Exp1AgeT) <- c("age","freq")
Exp1AgeT$age <- factor(Exp1AgeT$age, levels=c("18-29", "30-39", "40-49", "50-59", "+60"))
Fig1Exp1Age <- ggplot(Exp1Age, aes(x=age,color=age,fill=age))+theme_classic() + geom_bar(aes(),size=.1,show.legend=F) + geom_text(data=Exp1AgeT, aes(x=age,y=freq,label=freq), color="black", vjust=-0.2,size=3) + labs(x="Age",y="Number of participants") + coord_cartesian(ylim=c(0, 36))

# 5) Place
Exp1Place <- unique(dataExp1[,c("place","workerId")]);Exp1PlaceT <- as.data.frame(table(Exp1Place$place));names(Exp1PlaceT) <- c("place","freq")
place_levels <- c("north","south","overseas")
Exp1Place$place <- factor(Exp1Place$place, levels = place_levels)
Exp1PlaceT$place <- factor(Exp1PlaceT$place, levels = place_levels)
Fig1Exp1Place <- ggplot(Exp1Place, aes(x=place, color=place, fill= place)) + theme_classic() + geom_bar(aes(),size=.1,show.legend=F) + geom_text(data=Exp1PlaceT,aes(x=place,y=freq,label=freq), color="black", vjust=-0.2,size=3) + labs(x = "Place", y = "Number of participants") + coord_cartesian(ylim=c(0, 55))

# 6) Exposure to Māori
Exp1ME <- unique(dataExp1[,c("maoriExpo","workerId")]);Exp1ME$maoriExpo <- as.factor(Exp1ME$maoriExpo)
Exp1MET <- as.data.frame(table(Exp1ME$maoriExpo));names(Exp1MET) <- c("maoriExpo","freq")
Fig1Exp1ME <- ggplot(Exp1ME, aes(x=maoriExpo, color=maoriExpo, fill=maoriExpo))+theme_classic() + geom_bar(aes(),size=.1,show.legend=F) + geom_text(data=Exp1MET, aes(x=maoriExpo,y=freq,label=freq), color="black", vjust=-0.2,size=3)+labs(x="Level of exposure",y="Number of participants") + coord_cartesian(ylim=c(0, 28))

multiplot(Fig1Exp1Gender, Fig1Exp1Education, Fig1Exp1ML, Fig1Exp1Age, Fig1Exp1Place, Fig1Exp1ME, cols=2)
```

Overview of participants’ sociolinguistic profile in Experiment 1. Bars are labeled with their counts for each category.

## 1.3 Stimuli

The dataset contains responses to 131 real Māori words and 209 non-words. The properties of these stimuli are illustrated below.

```
stimuliExp1 = dataExp1 %>% 
  dplyr::select(word, type, macron, length, score, n.neighbors, mean.neighbor.logfreq, enteredResponse) %>% 
  group_by(word, type, macron, length, score, n.neighbors, mean.neighbor.logfreq) %>%
  summarise(
    mean.rating = mean(as.numeric(enteredResponse))
  ) %>%
  ungroup() %>%
  mutate(
    type = factor(type, levels=c("non", "real"), labels=c("Nonwords", "Words"))
  )

score_plot = stimuliExp1 %>%
  ggplot(., aes(x=score)) +
  geom_density(show.legend=F) +
  facet_grid(~ type) +
  labs(x="Phonotactic score", y="Density") +
  theme_bw() +
  theme(
    panel.grid = element_blank(),
    strip.text = element_text(size=12)
  )

macron_plot = stimuliExp1 %>%
  mutate(
    macron = factor(macron, levels=c(TRUE, FALSE), labels=c("Has macron(s)", "No macron(s)"))
  ) %>%
  count(across(c(type, macron)), .drop=FALSE) %>%
  ggplot(., aes(x=macron, y=n, fill=factor(macron))) +
  geom_col(show.legend=F) +
  geom_text(aes(label=n), vjust=-0.2) +
  facet_grid(~ type) +
  labs(x="Presence of macron", y="Number of stimuli") +
  scale_y_continuous(expand=expansion(add=c(0,45))) +
  theme_bw() +
  theme(
    panel.grid = element_blank(),
    strip.text = element_text(size=12)
  )

length_plot = stimuliExp1 %>%
  mutate(
    length = factor(length, levels=min(length):max(length))
  ) %>%
  count(across(c(type, length)), .drop=FALSE) %>%
  ggplot(., aes(x=length, y=n, fill=factor(length))) +
  geom_col(show.legend=F) +
  geom_text(aes(label=n), vjust=-0.2) +
  facet_grid(~ type) +
  labs(x="Length", y="Number of stimuli") +
  scale_y_continuous(expand=expansion(add=c(0,25))) +
  theme_bw() +
  theme(
    panel.grid = element_blank(),
    strip.text = element_text(size=12)
  )

neighbor_plot = stimuliExp1 %>%
  ggplot(., aes(x=n.neighbors)) +
  geom_density(show.legend=F) +
  facet_grid(~ type) +
  labs(x="Number of phonological neighbors", y="Density") +
  theme_bw() +
  theme(
    panel.grid = element_blank(),
    strip.text = element_text(size=12)
  )

ggarrange(score_plot, macron_plot, length_plot, neighbor_plot, ncol=1) 
remove(score_plot, macron_plot, length_plot, neighbor_plot)
```

Overview of word and non-word stimulus properties for Experiment 1. Distributions of non-word properties are shown in the left facets; distributions of word properties are shown in the right facets.

## 1.4 Summary of raw data

The plots below shows the mean response for each stimulus, broken down by type (word vs. nonword) and phonotactic score, presence of macron, length, and number of neighbors. As can be seen, words tend to get higher ratings than nonwords. Other apparent patterns include the tendency for stimuli (both words and nonwords) with high phonotactic scores or macrons to get higher ratings than stimuli with low phonotactic scores or no macrons.

```
score_plot = ggplot(stimuliExp1, aes(x=score, y=mean.rating, color=type, shape=type, label=word)) + 
  geom_point(size=3, alpha=0.2) + 
  geom_text(hjust=0.2, vjust=-0.7, check_overlap=TRUE, size=4) + 
  scale_shape_manual(values = c("Nonwords" = 19, "Words" = 17)) +  
  scale_color_manual(values = c("Nonwords" = "black", "Words" = "blue")) + 
  facet_grid( ~ type) + 
  labs(x= "Phonotactic score", y="Average rating") + 
  scale_x_continuous(expand=expansion(mult=0.1)) +
  ylim(1, 5.1) +
  theme_bw() + 
  theme(
    legend.position = "none", 
    legend.title = element_blank(),
    panel.background = element_blank(), 
    axis.text = element_text(size=13), 
    axis.title = element_text(size=13, face="bold"), 
    legend.text = element_text(size=12), 
    strip.text = element_text(size = 13, face="bold")
  )

macron_plot = ggplot(stimuliExp1, aes(x=macron, y=mean.rating, color=type, fill=type, label=word)) + 
  geom_violin(alpha=0.3, color=NA) + 
  geom_text(hjust=0.5, vjust=-0.7, check_overlap=TRUE, size=4) + 
  scale_color_manual(values = c("Nonwords" = "black", "Words" = "blue")) + 
  scale_fill_manual(values = c("Nonwords" = "black", "Words" = "blue")) + 
  facet_grid( ~ type) + 
  labs(x= "Presence of macron", y="Average rating") +
  ylim(1, 5.1) +
  theme_bw() + 
  theme(
    legend.position = "none", 
    legend.title = element_blank(),
    panel.background = element_blank(), 
    axis.text = element_text(size=13), 
    axis.title = element_text(size=13, face="bold"), 
    legend.text = element_text(size=12), 
    strip.text = element_text(size = 13, face="bold")
  )

length_plot = ggplot(stimuliExp1, aes(x=length, y=mean.rating, color=type, shape=type, label=word)) + 
  geom_point(size=3, alpha=0.2) + 
  geom_text(hjust=0.2, vjust=-0.7, check_overlap=TRUE, size=4) + 
  scale_shape_manual(values = c("Nonwords" = 19, "Words" = 17)) +  
  scale_color_manual(values = c("Nonwords" = "black", "Words" = "blue")) + 
  facet_grid( ~ type) + 
  labs(x= "Length (in phonemes)", y="Average rating") +
  scale_x_continuous(expand=expansion(mult=0.1)) +
  ylim(1, 5.1) +
  theme_bw() + 
  theme(
    legend.position = "none", 
    legend.title = element_blank(),
    panel.background = element_blank(), 
    axis.text = element_text(size=13), 
    axis.title = element_text(size=13, face="bold"), 
    legend.text = element_text(size=12), 
    strip.text = element_text(size = 13, face="bold")
  )

neighbor_plot = ggplot(stimuliExp1, aes(x=n.neighbors, y=mean.rating, color=type, shape=type, label=word)) + 
  geom_point(size=3, alpha=0.2) + 
  geom_text(hjust=0.2, vjust=-0.7, check_overlap=TRUE, size=4) + 
  scale_shape_manual(values = c("Nonwords" = 19, "Words" = 17)) +  
  scale_color_manual(values = c("Nonwords" = "black", "Words" = "blue")) + 
  facet_grid( ~ type) + 
  labs(x= "Number of phonological neighbors", y="Average rating") +
  scale_x_continuous(expand=expansion(mult=0.1)) +
  ylim(1, 5.1) +
  theme_bw() + 
  theme(
    legend.position = "none", 
    legend.title = element_blank(),
    panel.background = element_blank(), 
    axis.text = element_text(size=13), 
    axis.title = element_text(size=13, face="bold"), 
    legend.text = element_text(size=12), 
    strip.text = element_text(size = 13, face="bold")
  )

ggarrange(score_plot, macron_plot, length_plot, neighbor_plot, ncol=1) 
remove(score_plot, macron_plot, length_plot, neighbor_plot)
```

Average stimulus ratings for nonwords (left) and words (right), broken down by various stimulus properties.

## 1.5 Statistical analysis

Because the word and nonword stimuli differ along a number of dimensions (e.g. phonotactic score and neighborhood density), it is possible that the higher rating of words than nonwords is affected (in whole or in part) by these differences. To confirm that participants do indeed have knowledge of what is or is not a word of Māori, above and beyond effects of other attributes of stimuli, and that this knowledge is shared by participants in general, rather than held by a select few, we conduct (logit) ordinal mixed-effects regression. For an introduction to ordinal regression, see Section 5.1 of the Detailed Materials and Methods of Oh et al. (2020).

The predictors considered in the analysis are:

- `score`: Stimulus phonotactic score (centered)
- `type`: Stimulus type: word or nonword (binary factor)
- `macron`: Presence of a macron not found in English words (binary factor)
  - Note: we include this predictor both because it captures a visual component that is distinct from phonotactics, and because macrons indicate vowel length, which is not captured in our phonotactic scores following Oh et al. (2020)
- `length`: Stimulus length in phonemes (centered)
- `n.neighbors`: Number of phonological neighbors (centered)

We consider `score` and `type` to be the predictors of primary interest, following Oh et al. (2020), while `macron`, `length` and `n.neighbors` are control predictors.

We take a stepwise approach to model fitting, broken into stages in order to minimize the computational burden of estimating many random slopes. In the first stage, we consider fixed effects only (`clm`), with a model containing all two-way interactions between a predictor of primary interest (`score` and `type`) and a control predictor (`macron`, `length` and `n.neighbors`), as well as the interaction between the two predictors of primary interest. We remove terms one at a time if they do not make a significant contribution to the model’s explanatory power, assessed via Analysis of Deviance (using a type III test from the `anova` function), starting with the term with the least contribution. We do not consider the removal of a main effect until all interactions in which that predictor participates have also been removed. Once there are no more candidate terms for removal, we transition to the second stage, where we re-fit the model in a mixed-effects setting (`clmm`) with the minimal random effect structure of intercepts by participant and stimulus. We identify candidate fixed effects for removal in this setting if their coefficient is not significant in the model; after performing each removal, we confirm that it was justified via model comparison, using a likelihood ratio test with \(\alpha=0.1\). Once there are no more candidate terms for removal in this stage, we transition to the third stage, where we add by-participant random slopes for each remaining term in the model; in the event of failure of convergence, we try removing the correlations between slopes, starting with differentiating clusters whose predictors are in an interaction with each other. Other than as required for convergence, we do not remove predictors at the third stage.

The model table and partial effects plots are shown below. The main effect of `type` shows that participants rate real words higher than non-words. The main effect of `score` shows that participants rate nonwords higher the higher their phonotactic probability; however, the interaction between `score` and `type` shows that the same is not true for real words, where all words are rated equally high regardless of their phonotactics. Taken together, these results confirm the patterns suggested earlier by the high-frequency words studied by Panther et al. (under review): participants have phonotactic knowledge, and they recruit it to inform ratings when they do not recognize a stimulus, but do not need to recruit when they do recognize a stimulus (in this case, because it is a well known Māori word and perhaps a borrowing into New Zealand English). In addition, the main effect of `macron` shows that the visual presence of a macron is highly salient for participants and causes them to rate stimuli higher, consistent with earlier work (Oh et al., 2020). Finally, the main effect of `n.neighbors` shows that participants rate stimuli that closely resemble many real Māori words higher than those that resemble few words, supporting the conclusion of Oh et al. (2020) that they have a proto-lexicon of a large number of Māori words or word-parts, and suggesting that they can leverage this knowledge to do more than just judge phonotactic probability.

```
# Modify confidence ratings as discrete variables
dataExp1$enteredResponse <- as.factor(dataExp1$enteredResponse)

# # Stage 1: fixed effects only
# m1 <- clm(enteredResponse ~ (c.(score) + type) * (macron + c.(length) + c.(n.neighbors)) + c.(score):type, data=dataExp1)
# anova(m1, type="III") # Remove c.(score):c.(length)
# m2 <- update(m1, . ~ . - c.(score):c.(length))
# anova(m2, type="III") # Remove type:c.(length)
# m3 <- update(m2, . ~ . - type:c.(length))
# anova(m3, type="III") # Remove c.(score):c.(n.neighbors)
# m4 <- update(m3, . ~ . - c.(score):c.(n.neighbors))
# anova(m4, type="III") # No further candidate predictors for removal.
# 
# # Stage 2: mixed effects, random intercepts only.
# m5 <- clmm(enteredResponse ~ c.(score)*type + macron*(c.(score)+type) + c.(length) + c.(n.neighbors)*type + (1|workerId) + (1|word), data=dataExp1)
# summary(m5) # Removal candidate: c.(score):macron
# 
# m6 <- update(m5, . ~ . - c.(score):macron)
# anova(m5, m6) # Removal justified
# summary(m6) # Removal candidate: type:macron
# 
# m7 <- update(m6, . ~ . - type:macron)
# anova(m6, m7) # Removal justified
# summary(m7) # Removal candidate: c.(length)
# 
# m8 <- update(m7, . ~ . - c.(length))
# anova(m7, m8) # Removal justified
# summary(m8) # Removal candidate: type:c.(n.neighbors)
# 
# m9 <- update(m8, . ~ . - type:c.(n.neighbors))
# anova(m8, m9) # Removal justified
# summary(m9) # Removal candidate: c.(n.(neighbors))
# 
# m10 <- update(m9, . ~ . - c.(n.neighbors))
# anova(m9, m10) # p = 0.08065; removal NOT justified, so retain m9. No further candidates for removal
# 
# # Stage 3: mixed effects, random slopes
# m11a <- clmm(enteredResponse ~ c.(score) * type + macron + c.(n.neighbors) + (1 + c.(score) * type + macron + c.(n.neighbors) | workerId) + (1 | word), data=dataExp1) # Did not converge; try moving score*type slopes to not be correlated with the others
# 
# m11b <- clmm(enteredResponse ~ c.(score) * type + macron + c.(n.neighbors) + (1 + macron + c.(n.neighbors) | workerId) + (0 + c.(score)*type | workerId) + (1 | word), data=dataExp1)
# summary(m11b) # Converged
# 
# saveRDS(m11b, "model_exp1.rds")

mExp1 <- readRDS("model_exp1.rds")
clm_table(mExp1, caption="Model summary of confidence ratings. All numeric variables in this model are centered.")
```

Model summary of confidence ratings. All numeric variables in this model are centered.

|  | Parameter | Estimate | Std. Error | \(z\) | \(p\) |  |
| --- | --- | --- | --- | --- | --- | --- |
| Effects | score (centered) | 3.974 | 0.946 | 4.201 | <0.001 | \*\*\* |
|  | type = real | 4.734 | 0.241 | 19.608 | <0.001 | \*\*\* |
|  | macron = TRUE | 0.700 | 0.180 | 3.891 | <0.001 | \*\*\* |
|  | n.neighbors (centered) | 0.025 | 0.011 | 2.222 | 0.026 | \* |
|  | score (centered) × type = real | -3.212 | 1.577 | -2.037 | 0.042 | \* |
| Thresholds | 1|2 | -3.073 | 0.168 |  |  |  |
|  | 2|3 | -0.775 | 0.166 |  |  |  |
|  | 3|4 | 1.479 | 0.166 |  |  |  |
|  | 4|5 | 2.959 | 0.167 |  |  |  |

```
# 1) Phonotactic score * type
exp1_mean_1 <- clm_plotdat(mExp1, c("type", "score"), xlevels=list(score=25), type="mean") %>%
  mutate(
    type = fct_recode(type, "Nonword"="non", "Word"="real") %>% fct_relevel("Word"),
  ) %>%
  ggplot(., aes(x=score, y=pred, color=type, fill=type)) +
  geom_ribbon(aes(ymin=lci, ymax=uci), alpha=0.2, color=NA) +
  geom_line(size=1) +
  xlab("Phonotactic score") +
  ylab("Predicted mean rating") + 
  ylim(1, 5) +
  scale_color_manual(values = c("Nonword" = "black", "Word" = "blue")) +
  scale_fill_manual(values = c("Nonword" = "black", "Word" = "blue")) +
  theme_bw() + 
  theme(legend.position="none", panel.background = element_blank(), axis.text=element_text(size=13), axis.title=element_text(size = 13, face   = "bold"),legend.text=element_text(size=12), strip.text = element_text(size = 13, face="bold"))

exp1_dist_1 <- clm_plotdat(mExp1, c("type", "score"), xlevels=list(score=25), type="dist") %>%
  mutate(
    type = fct_recode(type, "Nonword"="non", "Word"="real") %>% fct_relevel("Word"),
  ) %>%
  rename("Rating" = response) %>%
  ggplot(., aes(x=score, y=pred, color=type, fill=type)) +
  geom_ribbon(aes(ymin=lci, ymax=uci), alpha=0.2, color=NA) +
  geom_line(size=1) +
  xlab("Phonotactic score") +
  ylab("Probability of rating") + 
  scale_color_manual(values = c("Nonword" = "black", "Word" = "blue")) +
  scale_fill_manual(values = c("Nonword" = "black", "Word" = "blue")) +
  facet_grid(. ~ Rating, labeller=label_both, switch="y") +
  ylim(0, 1) +  
  theme_bw() + 
  theme(legend.position="right", legend.title=element_blank(),panel.background = element_blank(), axis.text=element_text(size=13), axis.title=element_text(size = 13, face = "bold"),legend.text=element_text(size=12), strip.text = element_text(size = 13, face="bold"))

# 2) Macron
exp1_mean_2 <- clm_plotdat(mExp1, "macron", type="mean") %>%
  mutate(
    type="Word and\nNonword",
    macron = fct_recode(macron, "Yes"="TRUE", "No"="FALSE")
  ) %>%
  ggplot(., aes(x=macron, y=pred, color=type, fill=type)) +
  geom_point(size=4, position=position_dodge(width=0.2)) +
  geom_line(aes(x=as.integer(macron)), size=1, alpha=0.4, position=position_dodge(width=0.2)) +
  geom_errorbar(aes(ymin=lci, ymax=uci), size=1, width=0.3, position=position_dodge(width=0.2)) +
  xlab("Presence of macron") +
  ylab("Predicted mean rating") + 
  ylim(c(2.3, 5)) +
  scale_color_manual(values = c("Word and\nNonword" = "purple")) +
  scale_fill_manual(values = c("Word and\nNonword" = "purple")) +    
  theme_bw() + 
  theme(plot.title = element_text(hjust = 0.5, size=15),legend.position="none",legend.title=element_blank(),panel.background = element_blank(), axis.text=element_text(size=13), axis.title=element_text(size = 13, face = "bold"),   legend.text=element_text(size=12), strip.text = element_text(size = 13, face="bold"))

exp1_dist_2 <- clm_plotdat(mExp1, "macron", type="dist") %>%
  mutate(
    type="Word and\nNonword",
    macron = fct_recode(macron, "Yes"="TRUE", "No"="FALSE")
  ) %>%
  ggplot(., aes(x=macron, y=pred, color=type, fill=type)) +
  geom_point(size=4, position=position_dodge(width=0.2)) +
  geom_line(aes(x=as.integer(macron)), size=1, alpha=0.4, position=position_dodge(width=0.2)) +
  geom_errorbar(aes(ymin=lci, ymax=uci), size=1, width=0.3, position=position_dodge(width=0.2)) +
  xlab("Presence of macron") +
  ylab("Probability of rating") + 
  scale_color_manual(values = c("Word and\nNonword" = "purple")) +
  scale_fill_manual(values = c("Word and\nNonword" = "purple")) +  
  facet_grid(. ~ response, labeller=as_labeller(function(x) str_c("Rating: ", x))) +
  ylim(0, 1) +  
  theme_bw() + 
  theme(plot.title = element_text(hjust = 0.5, size=15),legend.position="right",legend.title=element_blank(), panel.background = element_blank(), axis.text=element_text(size=13), axis.title=element_text(size = 13, face= "bold"), legend.text=element_text(size=12), strip.text = element_text(size = 13, face="bold")) 

# 3) Number of neighbors
exp1_mean_3 <- clm_plotdat(mExp1, c("n.neighbors"), xlevels=list(n.neighbors=25), type="mean") %>%
  mutate(
    type = "Word and\nNonword"
  ) %>%
  ggplot(., aes(x=n.neighbors, y=pred, color=type, fill=type)) +
  geom_ribbon(aes(ymin=lci, ymax=uci), alpha=0.2, color=NA) +
  geom_line(size=1) +
  xlab("Number of neighors") +
  ylab("Predicted mean rating") + 
  ylim(1, 5) +
  scale_color_manual(values = c("Word and\nNonword" = "purple")) +
  scale_fill_manual(values = c("Word and\nNonword" = "purple")) +  
  theme_bw() + 
  theme(legend.position="none", panel.background = element_blank(), axis.text=element_text(size=13), axis.title=element_text(size = 13, face   = "bold"),legend.text=element_text(size=12), strip.text = element_text(size = 13, face="bold"))

exp1_dist_3 <- clm_plotdat(mExp1, c("n.neighbors"), xlevels=list(n.neighbors=25), type="dist") %>%
  mutate(
    type = "Word and\nNonword"
  ) %>%
  rename("Rating" = response) %>%
  ggplot(., aes(x=n.neighbors, y=pred, color=type, fill=type)) +
  geom_ribbon(aes(ymin=lci, ymax=uci), alpha=0.2, color=NA) +
  geom_line(size=1) +
  xlab("Number of neighbors") +
  ylab("Probability of rating") + 
  scale_color_manual(values = c("Word and\nNonword" = "purple")) +
  scale_fill_manual(values = c("Word and\nNonword" = "purple")) +  
  facet_grid(. ~ Rating, labeller=label_both, switch="y") +
  ylim(0, 1) +  
  theme_bw() + 
  theme(legend.position="right", legend.title=element_blank(),panel.background = element_blank(), axis.text=element_text(size=13), axis.title=element_text(size = 13, face = "bold"),legend.text=element_text(size=12), strip.text = element_text(size = 13, face="bold"))

ggarrange(exp1_mean_1, exp1_dist_1, exp1_mean_2, exp1_dist_2, exp1_mean_3, exp1_dist_3, labels=c("a.","","b.","","c.",""), ncol=2, widths=c(1,6))
remove(exp1_mean_1, exp1_dist_1, exp1_mean_2, exp1_dist_2, exp1_mean_3, exp1_dist_3)
```

Effect plots of: the interaction between phonotactic score and the distinction between non vs. real word stimuli (Fig.a); the presence of macrons (Fig.b); and number of phonological neighbors (Fig.c). Plots on the left show predicted mean ratings and plots on the right show predicted distributions over ratings.

Below, we report Variance Inflation Factors (VIFs) for the final model, which are calculated based on the extent to which each fixed effect predictor can be predicted from all other fixed effect predictors. Since this calculation is not dependent on the random effect structures, we perform it by applying the `vif()` function from the `car` package to a (fixed-effect) logit ordinal regression model with all of the fixed effects from the final ordinal regression model; since `vif()` is not applicable to models fit with the `ordinal` package at the time of writing, we instead apply it to a model fit with the `polr()` function from the `MASS` library. Large VIFs indicate multicollinearity, whereby one predictor is highly correlated with combinations of others, which decreases the extent to which inferences from the model can be trusted. For our model, all VIFs are below 2, which indicates low multicollinearity (VIFs above 5 are generally interpreted as potentially problematic, while VIFs above 10 are cause for serious concern).

```
clm_vif(mExp1, dataExp1, caption="Variance Inflation Factors for the final model from Experiment 1. All numeric variables in this model are centered.")
```

Variance Inflation Factors for the final model from Experiment 1. All numeric variables in this model are centered.

| Parameter | VIF |
| --- | --- |
| score (centered) | 1.702 |
| type | 1.308 |
| macron | 1.219 |
| n.neighbors (centered) | 1.211 |
| score (centered) × type | 1.728 |

## 1.6 Investigation of individual differences

Given the suggestion that phonotactic sensitivity loses its relevance for participants who actually know the words in the experiment, we now ask whether the patterns we see hold in general or whether they are different for people with different degrees of apparent knowledge of Māori.

To measure a participant’s apparent knowledge of Māori, we use \(d^\prime\) (d-prime), which indicates their sensitivity to real words vs. nonwords in the experiment. \(d^prime\) is the difference between a participant’s \(z\)-scored *hit rate* (proportion of real words given a rating of 4 or 5) and their \(z-scored\) *false alarm rate* (proportion of nonwords given a rating of 4 or 5); thus, participants with high \(d^\prime\) give many words and few nonwords a rating of 4 or 5, while participants with low \(d^\prime\) give fewer words and more nonwords this rating (but still give a higher proportion of words than nonwords a rating of 4 or 5, due to our exclusion of participants with a negative \(d^\prime\)). The formula for \(d^\prime\) is as follows, where the function \(z\left(\cdot\right)\) uses the function `qnorm()` in `R`:

\[
d^\prime = z\left(\frac{\text{# real words rated 4 or 5}}{\text{total # real words seen}}\right) - z\left(\frac{\text{# nonwords rated 4 or 5}}{\text{total # nonwords seen}}\right)
\]

In this formulation, \(d^\prime\) is undefined if a participant either gives *every* real word stimulus a 4 or 5 rating (correctly identifying every real word), or if they give *no* nonword stimulus a 4 or 5 rating (correctly rejecting every nonword). In our dataset, 5 participants have undefined \(d^\prime\)s (4 who correctly identified every real word, and 1 who correctly rejected every nonword). These participants are not included in the analysis that follows.

The distribution of \(d^\prime\) values across participants is shown in the figure below.

```
dataExp1 %>%
  filter(is.finite(dprime)) %>%
  group_by(workerId) %>%
  summarise(dprime = first(dprime)) %>%
  ggplot(., aes(x=dprime)) +
  geom_histogram(binwidth=0.1) +
  theme_bw() +
  theme(
    panel.background = element_blank(),
    axis.text = element_text(size=12), 
    axis.title = element_text(size = 14)
  )
```

Distribtion of d-prime values across participants in Experiment 1.

From the definition of \(d^\prime\), it follows that sensitivity to the `type` variable in Experiment 1 will have increased with \(d^\prime\): participants with high \(d^\prime\) will have given higher ratings to real words and lower ratings to nonwords, on average, than participants with low \(d^\prime\). \(d^\prime\) might therefore be thought of as capturing *how much* Māori word knowledge participants have. In this analysis, we are interested in whether participants with different degrees of word knowledge show different degrees of sensitivity to phonotactic `score`, presence of a `macron`, and `n.neighbors`. Thus, we build on the previous ordinal regression model to allow every term to interact with \(d^\prime\), and we investigate the nature of these interactions.

To avoid unnecessary computational burden, we follow the same strategy as previously, removing fixed effects from the model in stages. Note that, because our focus here is on the interactions with \(d^\prime\), at each stage we only consider removing terms that involve \(d^\prime\) as an interactant. In the first stage, we start with fixed effects only (`clm`), where we take all of the fixed effects that remained in the previous model, and allow each of them to interact with \(d^\prime\). We remove terms that do not contribute significantly to model fit, as assessed by Analysis of Deviance. In the second stage, we transition to a basic mixed effects model (`clmm`), adding random intercepts by participant and word. We identify potential removal candidates based on their \(p\)-value in the model summary, and confirm they can be removed without damaging model fit by a likelihood comparison test. In the third stage, we add random slopes, in the same structure as previously identified; because \(d^\prime\) is a participant-level variable, we add it as a by-word slope, and do not include random effects for any interactions involving \(d^\prime\). In the event of convergence issues, we consider making the by-word \(d^\prime\) slope uncorrelated with the word random intercept, and then consider dropping it altogether. Other than as required for convergence, we do not remove predictors at the third stage.

The summary of the model is shown below, together with partial effect plots. As expected from the definition of \(d^\prime\), participants with a lot of Māori word knowledge (high \(d^\prime\)) show a higher degree of separation between ratings for words and nonwords than participants with less Māori word knowledge (low \(d^\prime\)). But beyond this simple effect, there are many differences in degree of sensitivity to other factors as well: as a participant’s word knowledge increases, they make less use of phonotactic score in rating nonwords, less use of the presence of macrons, and more use of phonological neighbors. In sum, this suggests that a higher degree of word knowledge allows a participant to use more *specific* and *explicit* knowledge in this task: rather than using presence of a macron as a proxy, or a sort of generalized “Māori-like-ness” offered by phonotactic score, they make their ratings based on confident awareness of the existence of a word where possible, and based in part on awareness of specific highly-similar words (phonological neighbors) in other cases.

```
# Modify confidence ratings as discrete variables
dataExp1$enteredResponse <- as.factor(dataExp1$enteredResponse)

# Discard participants with infinite dprime
dataExp1DprimeFinite <- dataExp1[is.finite(dataExp1$dprime),]

# # Stage 1: fixed effects
# m1 <- clm(enteredResponse ~ c.(dprime) * (c.(score) * type + macron + c.(n.neighbors)), data=dataExp1DprimeFinite)
# anova(m1, type="III") # Remove c.(dprime):c.(score):type
# m2 <- update(m1, . ~ . - c.(dprime):c.(score):type)
# anova(m2, type="III") # No further removal candidates
# 
# # Stage 2: mixed effects, random intercepts only
# m3 <- clmm(enteredResponse ~ c.(dprime) * (c.(score) + type + macron + c.(n.neighbors)) + c.(score):type + (1 | workerId) + (1 | word), data=dataExp1DprimeFinite)
# summary(m3) # No removal candidates among dprime interaction terms
# 
# # Stage 3: mixed effects, random slopes (inheriting uncorrelated slopes from earlier model, and adding dprime as a slope by word)
# m4a <- clmm(enteredResponse ~ c.(dprime) * (c.(score) + type + macron + c.(n.neighbors)) + c.(score):type + (1 + macron + c.(n.neighbors) | workerId) + (0 + c.(score)*type | workerId) + (1 + dprime | word), data=dataExp1DprimeFinite) # Did not converge; try removing the correlation between the by-word dprime slope and intercept
# 
# m4b <- clmm(enteredResponse ~ c.(dprime) * (c.(score) + type + macron + c.(n.neighbors)) + c.(score):type + (1 + macron + c.(n.neighbors) | workerId) + (0 + c.(score)*type | workerId) + (1 | word) + (0 + dprime | word), data=dataExp1DprimeFinite)
# summary(m4b) # Did not converge; try removing dprime slope
# 
# m4c <- clmm(enteredResponse ~ c.(dprime) * (c.(score) + type + macron + c.(n.neighbors)) + c.(score):type + (1 + macron + c.(n.neighbors) | workerId) + (0 + c.(score)*type | workerId) + (1 | word), data=dataExp1DprimeFinite)
# summary(m4c) # Converged
# 
# saveRDS(m4c, "model_exp1_dprime.rds")

mExp1Dprime <- readRDS("model_exp1_dprime.rds")
clm_table(mExp1Dprime, caption="Model summary of confidence ratings with participants' d'. All numeric variables in this model are centered.")
```

Model summary of confidence ratings with participants’ d’. All numeric variables in this model are centered.

|  | Parameter | Estimate | Std. Error | \(z\) | \(p\) |  |
| --- | --- | --- | --- | --- | --- | --- |
| Effects | dprime (centered) | -0.321 | 0.175 | -1.832 | 0.067 | . |
|  | score (centered) | 4.039 | 0.950 | 4.252 | <0.001 | \*\*\* |
|  | type = real | 4.625 | 0.196 | 23.621 | <0.001 | \*\*\* |
|  | macron = TRUE | 0.693 | 0.176 | 3.932 | <0.001 | \*\*\* |
|  | n.neighbors (centered) | 0.025 | 0.011 | 2.234 | 0.025 | \* |
|  | dprime (centered) × score (centered) | -0.755 | 0.348 | -2.169 | 0.030 | \* |
|  | dprime (centered) × type = real | 1.943 | 0.153 | 12.735 | <0.001 | \*\*\* |
|  | dprime (centered) × macron = TRUE | -0.586 | 0.107 | -5.468 | <0.001 | \*\*\* |
|  | dprime (centered) × n.neighbors (centered) | 0.024 | 0.006 | 4.026 | <0.001 | \*\*\* |
|  | score (centered) × type = real | -3.174 | 1.583 | -2.005 | 0.045 | \* |
| Thresholds | 1|2 | -3.058 | 0.157 |  |  |  |
|  | 2|3 | -0.725 | 0.155 |  |  |  |
|  | 3|4 | 1.464 | 0.155 |  |  |  |
|  | 4|5 | 2.937 | 0.156 |  |  |  |

Effect plots of the interaction between dprime and: the interaction between phonotactic score and the distinction between non vs. real word stimuli (Fig.a); the presence of macrons (Fig.b); and number of phonological neighbors (Fig.c). Plots on the left show predicted mean ratings and plots on the right show predicted distributions over ratings; top panels in each case show predictions for a high-dprime participant (dprime=3; 93rd percentile in our data), while bottom panels show predictions for a low-dprime participant (dprime=1; 5th percentile in our data). Note that dprime was modeled as a continuous variable, and has been broken into dichotomous values here for illustrative purposes only.

Below, we report Variance Inflation Factors (VIFs) for the final model, calculated as previously described. All VIFs are below 2.5, which indicates low multicollinearity.

```
clm_vif(mExp1Dprime, dataExp1DprimeFinite, caption="Variance Inflation Factors for the final individual differences model from Experiment 1. All numeric variables in this model are centered.")
```

Variance Inflation Factors for the final individual differences model from Experiment 1. All numeric variables in this model are centered.

| Parameter | VIF |
| --- | --- |
| dprime (centered) | 2.262 |
| score (centered) | 1.687 |
| type | 1.387 |
| macron | 1.223 |
| n.neighbors (centered) | 1.242 |
| dprime (centered) × score (centered) | 1.389 |
| dprime (centered) × type | 1.848 |
| dprime (centered) × macron | 1.652 |
| dprime (centered) × n.neighbors (centered) | 1.232 |
| score (centered) × type | 1.751 |

# 2 Experiment 2

Experiment 1 confirmed the findings of Oh et al. (2020) and Panther et al. (under review): non-Māori-speaking New Zealanders are able to distinguish real Māori words from Māori-like nonwords, and their ratings in this task are affected by factors that depend on knowledge of a larger set of words (phonotactic score and number of phonological neighbors), as well as the presence of a visually salient macron. In particular, Experiment 1 extended on these previous studies to show that the effects of such factors persist even when participants are tasked with recognizing potential borrowings, i.e. words that are highly salient through their frequency of use in both Māori and New Zealand English, but that their degree of use varies between words and nonwords, and between participants with different degrees of Māori word knowledge. This result suggests that, for at least some stimuli, at least some non-Māori-speaking New Zealanders recruit explicit knowledge in a way that circumvents the need for the application of implicit knowledge.

In Experiment 2, we ask what level of explicit knowledge of Māori words non-Māori-speaking New Zealanders actually have. Do they just know that the words exist, or do they also know how they are used? We also ask how the various factors that we saw have an effect on word identification in Experiment 1 affect the level of explicit knowledge that non-Māori-speaking New Zealanders develop. Do these factors facilitate knowledge of the meanings of words, in the same way that they facilitate recognition of the existence of words?

In Experiment 2, participants were presented with a series of words, one at a time, and asked to define them. The words were chosen from among the real word stimuli used in Experiment 1 that had consistently high ratings across participants, so that we could be sure that participants would be familiar with them. We hand-scored the definitions for accuracy, following a consistent protocol and discussing any areas of uncertainty. Accuracy scores were simplified and converted to a binary variable (correct or incorrect) for the purposes of the present analysis.

## 2.1 Data

### 2.1.1 Exclusions

For Experiment 2, we excluded:

- 9 participants who indicated above-basic ability to speak or comprehend te reo Māori (i.e. rated greater than 2 on a scale of 0-5 for either speaking or comprehension);
- 1 participant who learned their English outside NZ and have been living outside NZ for more than 2 years;
- 1 participant who gave joke definitions;
- all responses to the stimulus *whanau*, which was supposed to be *whānau* (with a macron).

The exclusion process for Experiment 2 is detailed in the code below.

```
# Loading the data to filter out participants
dataExp2 <- read.delim("./dataAnonNotFilteredExp2_added.txt", sep ="\t", header = TRUE, encoding="UTF-8")

# Remove 9 participants whose speakMaori or compMaori is equal to or above 3
rmParticipant1Exp2 <- unique(dataExp2[dataExp2$speakMaori >= 3 | dataExp2$compMaori >= 3,]$workerId) 
# 1b817b93 f44d2e0f 2d528dd7 90e5e8cd aa592fef a95bbe09 35ace421 b3f52c92 e7a6cb02
dataExp2 <- dataExp2[!dataExp2$workerId %in% rmParticipant1Exp2,]

# Remove one participant who did not learn their English in NZ and have been living overseas for more than two years (duration == "long")
summaryExp2WorkerId <- unique(dataExp2[,c("workerId","firstLangCountry","place","duration")])
EngNotInNZExp2 <- summaryExp2WorkerId[!summaryExp2WorkerId$firstLangCountry=="NZ",]
rmParticipant2Exp2 <- unique(EngNotInNZExp2[EngNotInNZExp2$place=="overseas",]$workerId) 
# 1e48b18a
dataExp2 <- dataExp2[!dataExp2$workerId %in% rmParticipant2Exp2,]

# Detect participant whose median reactionTime is shorter than 2*SD below the mean of all participants
median_RT <- aggregate(dataExp2$reactionTime, by=list(dataExp2$workerId), median)
names(median_RT) <- c("workerId","median")
cut <- mean(median_RT$median)-2*sd(median_RT$median)
# median_RT[!median_RT$median > cut,]$workerId # None detected!

# Remove a participant with joke answers
dataExp2 <- dataExp2[!dataExp2$workerId=="eaed6b4d",]

# Check the total number of usable participants for Exp2
# length(unique(dataExp2$workerId)) # 123

# Add a column to indicate the presence of macrons
dataExp2$macron <- FALSE
dataExp2[grepl("\U0101|\U0113|\U012B|\U014D|\U016B",dataExp2$word),]$macron <- TRUE
dataExp2$macron <- as.factor(dataExp2$macron)

# Remove rows containing a word "whanau"
dataExp2 <- dataExp2[!dataExp2$word=="whanau",]
```

### 2.1.2 Dataset structure

The data is structured as follows:

- *workerId* is the unique ID for each participant.
- *definition* is each participant’s entered definition for each stimulus.
- *coding* is the marking for each definition.
- *correct* is binary: either the definition is correct (TRUE) or incorrect (FALSE).
- *confidence* is each participant’s confidence rating for each definition. (on a scale from 0 to 5).
- *familiarity* is the average rating for each Māori word obtained from 101 NMS New Zealanders in Experiment 1.
- *familiarity.latent* is the prediction from the model (without \(d^\prime\)) of Experiment 1 for the average rating for each Māori word, in the latent space. It is a version of `familiarity` that is transformed to be approximately linearly distributed, and that accounts for the influence of individual participants, neither of which are true of the previous `familiarity` variable.
- *reactionTime* is the reaction time for each rating (seconds).
- *length* is the phoneme length of each stimulus.
- *word* is the stimulus used for the rating.
- *speakMaori* is each participant’s report of how well they can speak Māori (on a scale from 0 to 5).
- *compMaori* is each participant’s report of how well they can understand/read Māori (on a scale from 0 to 5).
- *maoriProf* is the sum of quantified response for speakMaori and compMaori (participant Māori proficiency).
- *age* is the age group for each participant.
- *gender* is the gender reported by each participant.
- *ethnicity* is categorized into binary answers, either Māori (M) or non Māori (non M).
- *education* is each participant’s highest level of education.
- *children* is each participant’s report of whether they have had any children who have attended preschool or primary school in New Zealand in the past five years.
- *maoriList* is each participant’s basic knowledge of Māori (with a scale ranging from 0 to 9).
- *place* is each participant’s current place of living (3 levels: NZ North Island, NZ South Island, or Overseas).
- *duration* is each participant’s time living in their current place (2 levels: long is > 2 years; short is =< 2 years).
- *firstLang* is each participant’s first language.
- *firstLangCountry* is the country where each participant learned their first language.
- *anyOtherLangs* is any other languages each participant reports speaking.
- *hawaii* is the binary response to the question whether participants have lived in Hawaii.
- *anyPolynesian* is the binary response to the question whether participants know any Polynesian such as Hawaiian, Tahitian, Sāmoan, or Tongan.
- *whichPolynesian* is the information regarding participants’ knowledge of Polynesian languages, if they know any.
- *impairments* is the answer to the question whether participants have a history of any speech or language impairments.
- *maoriExpo* is each participant’s level of exposure to Māori (with a scale ranging from 0 to 10).
- *score* is the phonotactic score based on the 1,629 most frequent morph types derived from all words in the dictionary, normalized by the phonemic length of stimuli, ignoring vowel length distinctions, assuming that participants are attempting to parse stimuli into morphs.
- *n.neighbors* is the the number of words (from the Māori dictionary) that can be reached by adding, deleting, or substituting one phoneme in each stimulus.
- *mean.neighbor.logfreq* is the frequency-weighted phonological neighbourhood density.
- *macron* indicates whether or not the word includes a macron, which orthgraphically indicates a long vowel. We include the presence of macrons in our analysis because it is visually salient, but is not otherwise reflected in terms such as phonotactic score.

## 2.2 Participants

After exclusions, we were left with data from 123 participants. A summary of the demographics of the participants is presented below.

Overview of participants’ sociolinguistic profile in Experiment 2. Bars are labeled with their counts for each category.

## 2.3 Stimuli

The dataset contains responses to 117 real Māori words, constituting all words from Experiment 1 that were given a mean rating of at least 4, except for *Aotearoa* (‘New Zealand’), *Māori* (‘New Zealander of indigenous descent’), and *Pākehā* (‘New Zealander of European descent’), which are highly salient words that all New Zealanders can be expected to be able to define.

For each word, we calculated the predicted mean response to that word in Experiment 1, using the mixed-effects model from Experiment 1 (without \(d^\prime\)). We kept this prediction in the latent space assumed by logit ordinal regression, to avoid inducing artificial nonlinearities. This number is the `familiarity.latent` of the word. Note that familiarity reflects a combination of “actual” familiarity with “induced” familiarity through factors such as phonotactic probability, presence of a macron, and number of phonological neighbors.

Distributions of the properties of these stimuli are shown below:

```
stimuliExp2 = dataExp2 %>% 
  dplyr::select(word, macron, length, score, n.neighbors, mean.neighbor.logfreq, familiarity.latent, correct) %>% 
  group_by(word, macron, length, score, n.neighbors, mean.neighbor.logfreq, familiarity.latent) %>%
  summarise(
    prop.correct = mean(correct)
  ) %>%
  ungroup()

familiarity_plot = stimuliExp2 %>%
  ggplot(., aes(x=familiarity.latent)) +
  geom_density(show.legend=F) +
  labs(x="Familiarity", y="Density") +
  theme_bw() +
  theme(
    panel.grid = element_blank(),
    strip.text = element_text(size=12)
  )

score_plot = stimuliExp2 %>%
  ggplot(., aes(x=score)) +
  geom_density(show.legend=F) +
  labs(x="Phonotactic score", y="Density") +
  theme_bw() +
  theme(
    panel.grid = element_blank(),
    strip.text = element_text(size=12)
  )

macron_plot = stimuliExp2 %>%
  mutate(
    macron = factor(macron, levels=c(TRUE, FALSE), labels=c("Has macron(s)", "No macron(s)"))
  ) %>%
  count(across(c(macron)), .drop=FALSE) %>%
  ggplot(., aes(x=macron, y=n, fill=factor(macron))) +
  geom_col(show.legend=F) +
  geom_text(aes(label=n), vjust=-0.2) +
  labs(x="Presence of macron", y="Number of stimuli") +
  scale_y_continuous(expand=expansion(add=c(0,45))) +
  theme_bw() +
  theme(
    panel.grid = element_blank(),
    strip.text = element_text(size=12)
  )

length_plot = stimuliExp2 %>%
  mutate(
    length = factor(length, levels=min(length):max(length))
  ) %>%
  count(across(c(length)), .drop=FALSE) %>%
  ggplot(., aes(x=length, y=n, fill=factor(length))) +
  geom_col(show.legend=F) +
  geom_text(aes(label=n), vjust=-0.2) +
  labs(x="Length", y="Number of stimuli") +
  scale_y_continuous(expand=expansion(add=c(0,25))) +
  theme_bw() +
  theme(
    panel.grid = element_blank(),
    strip.text = element_text(size=12)
  )

neighbor_plot = stimuliExp2 %>%
  ggplot(., aes(x=n.neighbors)) +
  geom_density(show.legend=F) +
  labs(x="Number of phonological neighbors", y="Density") +
  theme_bw() +
  theme(
    panel.grid = element_blank(),
    strip.text = element_text(size=12)
  )

ggarrange(familiarity_plot, score_plot, macron_plot, length_plot, neighbor_plot, ncol=3) 
remove(familiarity_plot, score_plot, macron_plot, length_plot, neighbor_plot)
```

Overview of stimulus properties for Experiment 2.

## 2.4 Summary of raw data

The plots below show the accuracy rate (proportion correct definitions) for each stimulus, broken down by familiarity, phonotactic score, presence of macron, length, and number of neighbors. It is clear that words that were recognized more consistently in Experiment 1 are also defined more accurately in Experiment 2; however, it is also clear that participants’ generally struggle to accurately define a large proportion of these words, even though they recognize them readily as real Māori words. It is difficult to see clear effects of aspects other than familiarity, other than perhaps for the presence of macrons: participants seem less able to accurately define words with macrons than words without macrons. This may in part reflect the fact that, in terms of *actual word knowledge*, the familiarity threshold for including words with macrons in the experiment was effectively lower than that for words without macrons, since macrons inflated the ratings that participants gave in Experiment 1, and it was on the basis of these ratings that stimuli were selected for this experiment.

```
familiarity_plot = ggplot(stimuliExp2, aes(x=familiarity.latent, y=prop.correct, label=word)) + 
  geom_point(size=3, alpha=0.2, shape=17, color="blue") + 
  geom_text(hjust=0.2, vjust=-0.7, check_overlap=TRUE, size=4, color="blue") + 
  labs(x= "Exp1 familiarity (latent)", y="Accuracy rate") + 
  ylim(0, 1.05)+
  scale_x_continuous(expand=expansion(mult=0.2)) +
  theme_bw() + 
  theme(
    legend.position = "none", 
    legend.title = element_blank(),
    panel.background = element_blank(), 
    axis.text = element_text(size=13), 
    axis.title = element_text(size=13, face="bold"), 
    legend.text = element_text(size=12), 
    strip.text = element_text(size = 13, face="bold")
  )

score_plot = ggplot(stimuliExp2, aes(x=score, y=prop.correct, label=word)) + 
  geom_point(size=3, alpha=0.2, shape=17, color="blue") + 
  geom_text(hjust=0.2, vjust=-0.7, check_overlap=TRUE, size=4, color="blue") + 
  labs(x= "Phonotactic score", y="Accuracy rate") + 
  scale_x_continuous(expand=expansion(mult=0.1)) +
  ylim(0, 1.05)+
  theme_bw() + 
  theme(
    legend.position = "none", 
    legend.title = element_blank(),
    panel.background = element_blank(), 
    axis.text = element_text(size=13), 
    axis.title = element_text(size=13, face="bold"), 
    legend.text = element_text(size=12), 
    strip.text = element_text(size = 13, face="bold")
  )

macron_plot = ggplot(stimuliExp2, aes(x=macron, y=prop.correct, label=word)) + 
  geom_violin(alpha=0.3, fill="blue") + 
  geom_text(hjust=0.5, vjust=-0.7, check_overlap=TRUE, size=4, color="blue") + 
  labs(x= "Presence of macron", y="Accuracy rate") +
  ylim(0, 1.05)+
  theme_bw() + 
  theme(
    legend.position = "none", 
    legend.title = element_blank(),
    panel.background = element_blank(), 
    axis.text = element_text(size=13), 
    axis.title = element_text(size=13, face="bold"), 
    legend.text = element_text(size=12), 
    strip.text = element_text(size = 13, face="bold")
  )

length_plot = ggplot(stimuliExp2, aes(x=length, y=prop.correct, label=word)) + 
  geom_point(size=3, alpha=0.2, shape=17, color="blue") + 
  geom_text(hjust=0.2, vjust=-0.7, check_overlap=TRUE, size=4, color="blue") + 
  labs(x= "Length (in phonemes)", y="Accuracy rate") +
  xlim(1, 15) +
  ylim(0, 1.05)+ 
  theme_bw() + 
  theme(
    legend.position = "none", 
    legend.title = element_blank(),
    panel.background = element_blank(), 
    axis.text = element_text(size=13), 
    axis.title = element_text(size=13, face="bold"), 
    legend.text = element_text(size=12), 
    strip.text = element_text(size = 13, face="bold")
  )

neighbor_plot = ggplot(stimuliExp2, aes(x=n.neighbors, y=prop.correct, label=word)) + 
  geom_point(size=3, alpha=0.2, shape=17, color="blue") + 
  geom_text(hjust=0.2, vjust=-0.7, check_overlap=TRUE, size=4, color="blue") + 
  labs(x= "Number of phonological neighbors", y="Accuracy rate") +
  scale_x_continuous(expand=expansion(mult=0.1)) +
  ylim(0, 1.05)+
  theme_bw() + 
  theme(
    legend.position = "none", 
    legend.title = element_blank(),
    panel.background = element_blank(), 
    axis.text = element_text(size=13), 
    axis.title = element_text(size=13, face="bold"), 
    legend.text = element_text(size=12), 
    strip.text = element_text(size = 13, face="bold")
  )

ggarrange(familiarity_plot, score_plot, macron_plot, length_plot, neighbor_plot, ncol=2) 
remove(familiarity_plot, score_plot, macron_plot, length_plot, neighbor_plot)
```

Rate of accurate definitions per word, broken down by various stimulus properties.

## 2.5 Statistical analysis

To assess the extent to which various factors affected participants’ ability to accurately define words, we use mixed-effects logistic regression. In particular, we are interested to know if the factors that affected awareness / recognition of words in Experiment 1 – phonotactic score, presence of a macron, and number of phonological neighbors – offer additional advantages in being able to *define* words as well.

The predictors considered in the analysis are:

- `familiarity.latent`: Predicted mean rating for the stimulus in Experiment 1, in the latent space (centered)
- `score`: Stimulus phonotactic score (centered)
- `macron`: Presence of a macron not found in English words (binary factor)
  - Note: we include this predictor both because it captures a visual component that is distinct from phonotactics, and because macrons indicate vowel length, which is not captured in our phonotactic scores following Oh et al. (2020)
- `length`: Stimulus length in phonemes (centered)
- `n.neighbors`: Number of phonological neighbors (centered)

We observed in Experiment 1 that participants’ sensitivities to word properties differed with their degree of apparent Māori word knowledge. In a similar way, it is possible that participants’ sensitivity to word properties in being able to define words accurately in Experiment 2 will differ based on how widely known the word is. For this reason, we include interactions between `familiarity.latent` and the predictors that showed an effect in Experiment 1 – `score`, `macron`, and `n.neighbors`. We do not include additional interactions, since we have no theoretical reason to suspect them, and since the number of words for which we have data is relatively small.

We again take a stepwise approach to model fitting, with some slight changes due to the greater efficiency with which logistic regression models can be fit compared to (logit) ordinal regression. In the first stage, we consider a mixed-effects (`glmer`) model where the only random effects are intercepts by participant and word. We use this model to prune unnecessary interaction terms from the model, to avoid the explosion of computational effort they bring for estimating random slopes: we remove non-significant interaction terms one at a time, starting with the term with highest \(p\) value, confirming via a likelihood ratio test (\(\alpha=0.1\)) after each removal that the term did not make a significant contribution to the model’s explanatory power. We do not consider removing main effects at the first stage. Once there are no further candidate interaction terms to remove, we move to the second stage, where we add correlated by-participant random slopes for all remaining terms. In the event of convergence issues, we try removing correlations between random slopes before removing any slopes altogether. Other than as required for convergence, we do not remove predictors at the second stage.

The model summary table and partial effects plots are below. Only `familiarity.latent` has a significant effect in the final model: the accuracy with which a word could be defined was higher the more consistently and confidently that word could be recognized in Experiment 1, but was not otherwise significantly affected by other factors. The marginal effect of `macron`, whereby words with a macron were numerically less likely to be accurately defined than words without a macron, is likely an artifact of the fact that words with a macron received inflated ratings in Experiment 1 – and thus inflated `familiarity.latent` – due to the salience of the macron, rather than actual familiarity; it is also affected by the fact that almost all participants confused *mauī* (‘left (direction)’) with *Māui* (a demigod who is commonly known through myths).

In interpreting the results of Experiment 2, it is important to remember that participants’ ratings of words in Experiment 1 were affected by properties of those words, such as phonotactic score and phonological neighborhood density. Thus, while these properties do not appear to offer *additional* advantages in tying explicit semantic knowledge to wordforms, they do offer advantages in the logical precursors of building awareness and recognition of the wordforms in the first place.

The results also speak to an intriguing question raised in recent lines of work: where does the knowledge of phonotactic probability and phonological neighbors that bestows advantages in awareness and recognition come from? Traditionally in phonological theory, both phonotactic probability and neighborhood density are assumed to derive from generalization over the mental lexicon. Oh et al. (2020) showed that non-Māori-speaking New Zealanders are able to track Māori phonotactic probabilities surprisingly well, to an extent that is best modeled through generalization over the forms of some 1,500 frequent Māori words and word-parts. But the mental lexicon of non-Māori-speaking New Zealanders cannot be anywhere near this size, at least on an understanding of the term “mental lexicon” as a store of forms *together with associated semantic (and syntactic, etc.) knowledge*, because the results of Experiment 2 clearly show that participants do not have explicit semantic knowledge of many words at all. Experiment 2 thus further supports Oh et al.’s claim that non-Māori-speaking New Zealanders have a Māori *proto-lexicon*, consisting of implicit knowledge of a large number of forms without necessarily any semantic associations. This claim is further supported by the finding from Experiment 1 that participants are sensitive to phonological neighborhood density, since the calculation of phonological neighbors requires specific knowledge of entire wordforms, above and beyond the general awareness of phoneme transition probabilities that may be sufficient for calculating phonotactic probabilities.

```
# # Stage 1: mixed effects, intercepts only, removing interaction terms only
# m1 <-  glmer(correct ~ c.(familiarity.latent) * (c.(score) + macron + c.(n.neighbors)) + c.(length) + (1 | workerId) + (1 | word), family="binomial", data=dataExp2, control=glmerControl(optimizer="bobyqa", optCtrl=list(maxfun=1e6)))
# summary(m1) # Removal candidate: c.(familiarity.latent):macron
# m2 <- update(m1, . ~ . - c.(familiarity.latent):macron)
# anova(m1, m2, test="LRT") # Removal justified
# summary(m2) # Removal candidate: c.(familiarity.latent):c.(score)
# m3 <- update(m2, . ~ . - c.(familiarity.latent):c.(score))
# anova(m2, m3, test="LRT") # Removal justified
# summary(m3) # Removal candidate: c.(familiarity.latent):c.(n.neighbors)
# m4 <- update(m3, . ~ . - c.(familiarity.latent):c.(n.neighbors))
# anova(m3, m4, test="LRT") # Removal justified
# summary(m4) # All interactions removed; no further candidates for removal
# 
# # Stage 2: add by-participant random slopes
# m5 <- glmer(correct ~ c.(familiarity.latent) + c.(score) + macron + c.(n.neighbors) + c.(length) + (1 + c.(familiarity.latent) + c.(score) + macron + c.(n.neighbors) + c.(length) | workerId) + (1 | word), family="binomial", data=dataExp2, control=glmerControl(optimizer="bobyqa", optCtrl=list(maxfun=1e6)))
# summary(m5) # Boundary (singular) fit. Random slope for c.(n.neighbors) has extremely low variance: try making it uncorrelated
# 
# m5a <- glmer(correct ~ c.(familiarity.latent) + c.(score) + macron + c.(n.neighbors) + c.(length) + (1 + c.(familiarity.latent) + c.(score) + macron + c.(length) | workerId) + (0 + c.(n.neighbors) | workerId) + (1 | word), family="binomial", data=dataExp2, control=glmerControl(optimizer="bobyqa", optCtrl=list(maxfun=1e6)))
# summary(m5a) # Boundary (singular) fit. Random slope for c.(n.neighbors) is estimated to zero: remove
# 
# m5b <- glmer(correct ~ c.(familiarity.latent) + c.(score) + macron + c.(n.neighbors) + c.(length) + (1 + c.(familiarity.latent) + c.(score) + macron + c.(length) | workerId) + (1 | word), family="binomial", data=dataExp2, control=glmerControl(optimizer="bobyqa", optCtrl=list(maxfun=1e6)))
# summary(m5b) # Boundary (singular) fit. Random slope for c.(score) shows lowest correlations: try making it uncorrelated
# 
# m5c <- glmer(correct ~ c.(familiarity.latent) + c.(score) + macron + c.(n.neighbors) + c.(length) + (1 + c.(familiarity.latent) + macron + c.(length) | workerId) + (0 + c.(score) | workerId) + (1 | word), family="binomial", data=dataExp2, control=glmerControl(optimizer="bobyqa", optCtrl=list(maxfun=1e6)))
# summary(m5c) # No issues with fit
# 
# saveRDS(m5c, file = "model_exp2.rds")

mExp2 <- readRDS("model_exp2.rds")
logistic_table(mExp2, caption="Model summary of accuracy of participants' definitions. All numeric variables in this model are centered.")
```

Model summary of accuracy of participants’ definitions. All numeric variables in this model are centered.

| Parameter | Estimate | Std. Error | \(z\) | \(p\) |  |
| --- | --- | --- | --- | --- | --- |
| (Intercept) | 0.773 | 0.180 | 4.300 | <0.001 | \*\*\* |
| familiarity.latent (centered) | 1.068 | 0.083 | 12.837 | <0.001 | \*\*\* |
| score (centered) | 0.080 | 1.406 | 0.057 | 0.955 |  |
| macron = TRUE | -0.502 | 0.279 | -1.799 | 0.072 | . |
| n.neighbors (centered) | -0.029 | 0.020 | -1.413 | 0.158 |  |
| length (centered) | -0.106 | 0.079 | -1.342 | 0.179 |  |

```
# 1) familiarity
exp2_mean_1 <- Effect("familiarity.latent", mExp2, xlevels=list(familiarity.latent = 25)) %>%
  as_tibble() %>%
  ggplot(., aes(x=familiarity.latent, y=fit)) +
  geom_ribbon(aes(ymin=lower, ymax=upper), alpha=0.2, color=NA, fill="blue") +
  geom_line(size=1, color="blue") +
  xlab("Exp1 familiarity (latent)") +
  ylab("Predicted definition accuracy") + 
  ylim(0, 1) +
  theme_bw() + 
  theme(legend.position="none", panel.background = element_blank(), axis.text=element_text(size=13), axis.title=element_text(size = 13, face   = "bold"),legend.text=element_text(size=12), strip.text = element_text(size = 13, face="bold"))

# 2) Macron
exp2_mean_2 <- Effect("macron", mExp2) %>%
  as_tibble() %>%
  ggplot(., aes(x=macron, y=fit)) +
  geom_point(size=4, position=position_dodge(width=0.2), color="blue") +
  geom_line(aes(x=as.integer(macron)), size=1, alpha=0.4, position=position_dodge(width=0.2), color="blue") +
  geom_errorbar(aes(ymin=lower, ymax=upper), size=1, width=0.3, position=position_dodge(width=0.2), color="blue") +
  xlab("Presence of macron") +
  ylab("Predicted mean rating") + 
  ylim(0, 1) +
  theme_bw() + 
  theme(plot.title = element_text(hjust = 0.5, size=15),legend.position="none",legend.title=element_blank(),panel.background = element_blank(), axis.text=element_text(size=13), axis.title=element_text(size = 13, face = "bold"),   legend.text=element_text(size=12), strip.text = element_text(size = 13, face="bold"))

ggarrange(exp2_mean_1, exp2_mean_2, labels=c("a.","b."), ncol=2)
remove(exp2_mean_1, exp2_mean_2)
```

Effect plots of familiarity (Fig.a) and presence of a macron (Fig.b).

Below, we report Variance Inflation Factors (VIFs) for the final model, calculated via direct usage of the `vif()` function (i.e. without refitting the model, since `vif()` supports `glmer` model objects). All VIFs are below 3, which indicates limited multicollinearity.

```
vif(mExp2) %>%
  vif_table(caption="Variance Inflation Factors for the final model from Experiment 2. All numeric variables in this model are centered.")
```

Variance Inflation Factors for the final model from Experiment 2. All numeric variables in this model are centered.

| Parameter | VIF |
| --- | --- |
| familiarity.latent (centered) | 1.066 |
| score (centered) | 1.323 |
| macron | 1.141 |
| n.neighbors (centered) | 2.886 |
| length (centered) | 2.519 |

# 3 Supporting Information

## 3.1 Appendix A: Post-questionnaire

1. How well are you able to speak Māori?  
   \(\square\) Very well (I can talk about almost anything in Māori)  
   \(\square\) Well (I can talk about many things in Māori)  
   \(\square\) Fairly well (I can talk about some things in Māori)  
   \(\square\) Not very well (I can only talk about simple/basic things in Māori)  
   \(\square\) No more than a few words or phrases  
   \(\square\) Not at all
2. How well are you able to understand/read Māori?  
   \(\square\) Very well (I can understand almost anything said/written in Māori)  
   \(\square\) Well (I can understand many things said/written in Māori)  
   \(\square\) Fairly well (I can understand some things said/written in Māori  
   \(\square\) Not very well (I can only understand simple/basic things said/written in Māori)  
   \(\square\) No more than a few words or phrases  
   \(\square\) Not at all
3. Which age group do you belong to?  
   \(\square\) 18 - 29  
   \(\square\) 30 - 39  
   \(\square\) 40 - 49  
   \(\square\) 50 - 59  
   \(\square\) +60
4. Please state your gender:
5. Please state your ethnicity:
6. Your highest education is:  
   \(\square\) High school  
   \(\square\) Undergraduate degree  
   \(\square\) Graduate degree
7. How often do you think you are exposed to the Māori language in your daily life, by means of Māori radio, Māori TV, online media?  
   \(\square\) Less than once a year  
   \(\square\) Less than once a month  
   \(\square\) Less than once a week  
   \(\square\) Less than once a day  
   \(\square\) Multiple times a day
8. How often do you think you are exposed to Māori language in your daily life, in conversation at work, at home, in social settings?  
   \(\square\) Less than once a year  
   \(\square\) Less than once a month  
   \(\square\) Less than once a week  
   \(\square\) Less than once a day  
   \(\square\) Multiple times a day
9. In the past five years, have you had any children living with you who have attended preschool or primary school in New Zealand?  
   \(\square\) Yes  
   \(\square\) No
10. Please tick all boxes that apply.  
    \(\square\) I can give a mihi in Māori.  
    \(\square\) I can sing a few songs in Māori.  
    \(\square\) I can sing the NZ national anthem in Māori.  
    \(\square\) I know how to say some basic phrases (e.g. My name is…, I’m from…) in Māori.  
    \(\square\) I know how to say some commands (e.g. Sit down / Come here) in Māori.  
    \(\square\) I know how to say some greetings in Māori.  
    \(\square\) I know how to say some numbers in Māori.  
    \(\square\) I know how to say some body parts in Māori.  
    \(\square\) I know how to say some colors in Māori.
11. What region of New Zealand do you live in currently? (Please choose ``overseas" if you are living outside of New Zealand.)  
    \(\square\) Northland  
    \(\square\) Auckland  
    \(\square\) Waikato  
    \(\square\) Bay of Plenty  
    \(\square\) Gisborne  
    \(\square\) Hawke’s Bay  
    \(\square\) Taranaki  
    \(\square\) Wanganui  
    \(\square\) Manawatu  
    \(\square\) Wairarapa  
    \(\square\) Wellington  
    \(\square\) Nelson Bays  
    \(\square\) Marlborough  
    \(\square\) West Coast  
    \(\square\) Canterbury  
    \(\square\) Timaru - Oamaru  
    \(\square\) Otago  
    \(\square\) Southland  
    \(\square\) Overseas
12. How long have you been living there?
13. Please state your first language (the language you speak/use most of your time).
14. What country were you living in when you first learned this language?
15. Please list any other languages that you can speak fluently:
16. Have you ever lived in Hawaii?  
    \(\square\) Yes  
    \(\square\) No
17. Do you speak/understand any Polynesian languages such as Hawaiian, Tahitian, Sāmoan, or Tongan?  
    \(\square\) Yes  
    \(\square\) No
18. If you replied yes to question 17, please state the language you know.
19. Do you have a history of any speech or language impairments that you are aware of?  
    \(\square\) Yes \(\square\) No

## 3.2 Appendix B: Stimulus materials for Experiments

### 3.2.1 Experiment 1

The real word stimuli used in Experiment 1 are:  
ako; aoraki; aotearoa; aroha; atua; awa; haere mai; haka; hangi; hapū; hīkoi; hōhā; hoki; hongi; hope; hui; ihu; iti; iwa; iwi; kaha; kahurangi; kai; kai moana; kāinga; kaitiaki; kaiwhakahaere; kākāriki; kapa haka; karakia; karanga; karu; katoa; kaumātua; kaupapa; kāwanatanga; kia kaha; kia ora; koha; kōhanga; kōrero; koro; korowai; koru; kōwhai; kuia; kura; kurī; mahi; mana; manuhiri; māori; marae; matariki; mate; mauī; maunga; māwhero; mere; mihi; moana; moko; mokopuna; mōrena; motu; noho; nui; ono; ora; pai; pākehā; pango; papa; papatūānuku; poi; pokohiwi; pounamu; pōwhiri; puke; puku; rangatira; rangatiratanga; rangi; ranginui; reo; rima; ringaringa; roto; rua; tahi; taiaha; taihoa; tamariki; tāne; tāngata; tangata whenua; tangi; taniwha; taonga; tapu; taringa; teina; tekau; tēnā koe; tēnā koutou; tikanga; tiki; tohunga; toru; tuakana; tupuna; turituri; upoko; utu; waewae; waha; wāhi tapu; wahine; wai; waiata; waka; wānanga; waru; whaea; whakapapa; whakarongo; whāngai; whare; whenua; whero; whitu

The nonword stimuli used in Experiment 1 are:  
ahatiati; ahiahake; amu; ape; apēhia; arane; ario; eha; eko; haeo; hakaatū; hāno; hepaua; hepiti; hewe; hiamu; hingi; hiu; hoengaima; hoihoko; hōke; horetī; howaka; huengi; hūku; humo; hunge; hupū; ihiri; ikau; iko; inga; ingi; iniata; ino; ire; iru; kawaa; kāweroni; kawha; kawha kawha; kawha nia; kawha whani; kemoramo; kepi; kingiro; kitō; kōioromāpara; komekua; kōmuawhiu; kōua; kūhatapō; kupō; kūro; kūwhati; māheneketoa; makei; mamatōhī; mango; māorawau; māorua; mautāmu; māwi; meahua; mero; mie; mihea; mini; moapi; moeo; mōha; mōnga; mungi; mupati; naipu; nānga; natoi; neetia; nema; ngae; ngaena; ngapoto; ngawhāniti; ngehi; ngema; ngemetata; ngepa; ngoa; ngue; nguta; nia; nia ire; nia kawha; nia pukau; nia uti; nia whani; nia whihia; nito; nitumaotaha; nōitia; nopo; nue; nuhi; nure; nuti; pahapā; pāhāpāko; paihoui; pāuki; paurounu; pāwhi; peu; pewe; pie; pīhu; pikeko; pīngi; poraki; poraki pukau; pote; pukau; pukau nia; pume; pūno; puora; pūrawha; pūtio; pūwhi; rahue; rangu; rapeia; rapuko; reru; rowa; rowhaohi; rukō; rume; rumo; rūne; rungu; rupa; rupo; taetū; tāhuma; takamīa; tākapī; tāmarutō; taongirua; tapopa; tārorangī; tatūhe; tawhengawhi; teaori; temi; tetohe; tetoua; teu; tīahu; tie; tikaweneri; tīkīhiki; tikōha; tikū; tīpe; tīpo; tītā; titapa; titapa pukau; tiwhi; tohiāhia; toketi; touki; tuanapū; tūkeiati; tumeiroruare; tuwhe; uke; uko; unati; uro; uti; waemura; wawemiti; wehao; wereu; whaha; whāhu; whaiē; whakōiaweahua; whāngaki; whani; whani kawha; whani nia; whani poraki; whani titapa; whani whani; whataī; whehu; whenepōna; whengo; wheto; wheu; whihia; whihia nia; whuri; whutarirari; wikuruta; wura; wuri

### 3.2.2 Experiment 2

The stimuli used in Experiment 2 are:  
aoraki; aroha; atua; awa; haere mai; haka; hangi; hapū; hīkoi; hōhā; hoki; hongi; hui; iti; iwa; iwi; kaha; kahurangi; kai; kai moana; kāinga; kaitiaki; kākāriki; kapa haka; karakia; karanga; katoa; kaumātua; kaupapa; kāwanatanga; kia kaha; kia ora; koha; kōhanga; kōrero; koro; korowai; koru; kōwhai; kuia; kura; kurī; mahi; mana; manuhiri; marae; matariki; mauī; maunga; māwhero; mere; mihi; moana; moko; mokopuna; mōrena; motu; noho; nui; ono; ora; pai; papa; papatūānuku; poi; pounamu; pōwhiri; puke; puku; rangatira; rangatiratanga; rangi; ranginui; reo; rima; ringaringa; roto; rua; tahi; taiaha; taihoa; tamariki; tāne; tāngata; tangata whenua; tangi; taniwha; taonga; tapu; taringa; teina; tekau; tēnā koe; tēnā koutou; tikanga; tiki; tohunga; toru; tuakana; tupuna; utu; waewae; wāhi tapu; wahine; wai; waiata; waka; wānanga; waru; whaea; whakapapa; whakarongo; whāngai; whare; whenua; whero; whitu
